# Smartphone-Assisted Aerogel Colorimetric Sensor Array Coupled with Lightweight Deep Learning for Multi-Meat Freshness Monitoring

**DOI:** 10.3390/gels12100910

**Published:** 2026-10-09

**Authors:** Hanzhi Chen, Xiao Zhou, Caoyuan Zhang, Zengrui Wu, Bo Peng, Yingli Wang

**Affiliations:** 1 College of Engineering, Huazhong Agricultural University, Wuhan 430070, China; hanzhichen@webmail.hzau.edu.cn (H.C.); ruiw@webmail.hzau.edu.cn (Z.W.); 2 Ningbo Academy of Agricultural Sciences, Ningbo 315040, China; xiaozhou_2023@163.com; 3 School of Life and Health Sciences, Hubei University of Technology, Wuhan 430068, China; zcy9up@163.com

**Keywords:** meat freshness, colorimetric sensor array, aerogel, smartphone imaging, lightweight deep learning, TVB-N

## Abstract

Rapid assessment of chilled-meat freshness is constrained by conventional laboratory-based measurements. Although food-detection studies emphasize predictive performance, computational cost is reported less consistently. Here, we developed a 2 × 2 colorimetric sensor array based on a porous konjac glucomannan (KGM)/gelatin/starch/SiO_2_ aerogel substrate loaded with four pH-sensitive dyes. Sensor images were acquired with a smartphone under standardized conditions, and four dye-responsive regions of interest (ROIs) were manually extracted and assembled into 2 × 2 dye-grid images. Five lightweight models—Spot-CNN, ShuffleNetV2-0.5x, EfficientNet-Lite0, MobileNetV3-Small and Tiny-MLP—were evaluated for classifying beef and pork freshness as Fresh, Sub-fresh or Spoiled. During chilled storage, the sensor color responses showed broadly consistent temporal trends with total volatile basic nitrogen (TVB-N). Under the fixed group-level split, Spot-CNN achieved image-unit-level accuracies of 0.933 and 0.938 and macro-AUC values of 0.995 and 0.982 on the beef and pork test sets, respectively. Tiny-MLP contained 0.013 M parameters and required less than 0.001 G floating-point operations, demonstrating lower computational cost. These results establish a controlled-laboratory prototype integrating an aerogel colorimetric sensor array, standardized smartphone image processing and lightweight-model comparison for multi-meat freshness screening. Further validation across meat batches, smartphone devices, lighting conditions and deployment environments is required.

## 1. Introduction

Freshness deterioration in chilled meat is driven in part by microbial decomposition of proteins and other nitrogenous compounds, which generates volatile basic substances such as ammonia (NH_3_), dimethylamine (DMA) and trimethylamine (TMA) and reduces sensory acceptability. Physicochemical indicators, including total volatile basic nitrogen (TVB-N), total viable count (TVC) and pH, are commonly used to characterize spoilage progression [1]. However, conventional measurements generally require laboratory facilities, chemical reagents and trained personnel, which limits their use for rapid screening outside laboratory settings [2]. Intelligent packaging and visual sensing provide alternative routes for communicating quality changes: active intelligent packaging has combined preservation and freshness monitoring for beef [3], and pH-sensitive films have been used to monitor pork [4] and seafood [5]. Smartphone-readable optical sensing of spoilage-related biogenic amines has also been demonstrated for food-freshness evaluation [6]. These approaches motivate an integrated strategy that converts sensor responses into standardized, interpretable freshness outputs.

Smartphone imaging offers a practical route for acquiring colorimetric sensor responses. Smartphone-integrated sensors can convert images into color descriptors such as red–green–blue (RGB), CIE L*a*b* (Lab) and the corresponding color difference (ΔE*ab) [7], which can subsequently be analyzed using machine-learning models for quantitative prediction or graded classification [8]. A mobile dual-response colorimetric array coupled with deep learning has also been reported for freshness assessment of aquatic products [9]. A multi-indicator colorimetric sensor array coupled with a smartphone application has been used to classify the freshness of beef, lamb and buffalo, with freshness classes defined by TVB-N levels [10]. Standardized smartphone photographs combined with a modified ResNeXt convolutional neural network (CNN) have also been used to classify large-yellow-croaker freshness at 12 and 24 h intervals [11]. Smartphone-embedded machine learning has also been evaluated across multiple smartphone brands and illumination conditions for on-site food-spoilage monitoring [12]. A recent review of smartphone-based meat-freshness labels further highlighted image standardization and label stability as practical considerations for deployment [13]. Collectively, these studies demonstrate the analytical potential of smartphone-assisted colorimetry. However, reliable image-based analysis still requires control of acquisition conditions and a consistent representation of the sensing regions.

The sensor substrate determines how dyes are immobilized and how volatile compounds contact the sensing phase. Aerogels provide a three-dimensional porous framework with relatively high porosity and specific surface area [14], and ammonia-responsive aerogel sensors have been used for non-destructive freshness monitoring [15]. Recent work has further emphasized the role of structure-engineered porous sensors in volatile-amine detection [16]. For low-temperature and high-humidity environments, bilayer hydrogel architectures [17] and hydrophobic nano-SiO_2_ coatings [18] have been explored to protect or stabilize the sensing interface. Chitosan-based [19] and anthocyanin-functionalized [20] aerogels also illustrate the adaptability of porous colorimetric substrates for meat-freshness monitoring. Recent studies have also demonstrated pH-responsive polysaccharide and gelatin-based gels for colorimetric monitoring of beef and poultry freshness [21,22]. Nevertheless, a responsive material alone does not ensure standardized image inputs: automatic white balance, imaging distance and device-dependent color rendering can alter smartphone images. Accordingly, the present study treated extraction of the four dye-responsive regions and their fixed ordering as explicit constraints in the image-construction workflow.

Colorimetric sensor arrays generate cross-reactive patterns that can be analyzed using models capable of learning nonlinear combinations of dye responses. Smartphone-derived colorimetric changes have been coupled with an artificial neural network for chicken-meat spoilage detection [23]. Deep-learning approaches have been applied to colorimetric and fluorescent arrays for raw-meat freshness assessment [24], while olfactory visualization sensors coupled with deep learning have been used for fruit-ripeness recognition [25]. Internet of Things (IoT)-based sensing systems have also been evaluated for meat-freshness classification [26]. These studies support learned pattern recognition, but computational cost and specimen-group validation are not always reported together with classification performance. The present study therefore compared five lightweight image-based and feature-based models on the same aerogel colorimetric-array datasets and evaluated predictive metrics, theoretical computational cost and partition-related variability.

**Figure 1:**
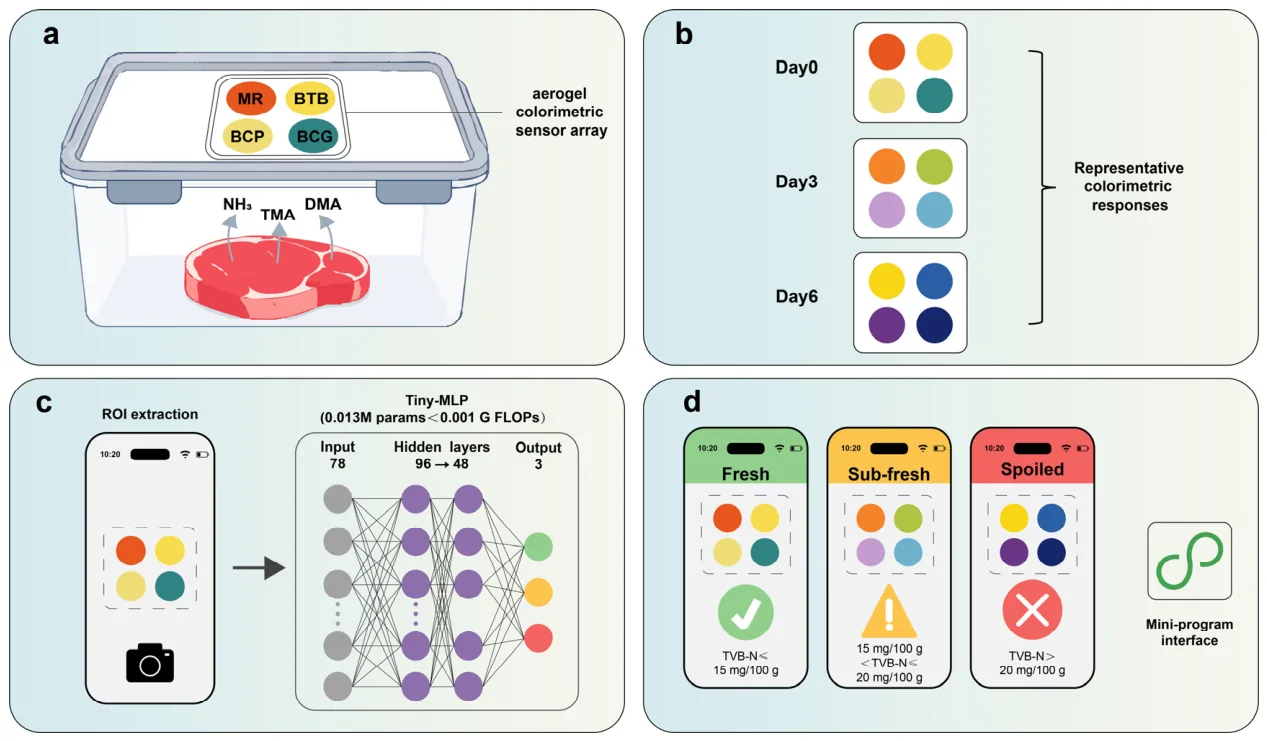
Smartphone-oriented workflow for meat-freshness monitoring. (**a**) Aerogel colorimetric sensor array exposed to meat volatiles in a sealed container; (**b**) representative color responses during chilled storage; (**c**) smartphone imaging, manual extraction of four dye ROIs, and Tiny-MLP analysis; (**d**) prototype display of Fresh, Sub-fresh, and Spoiled results. The three classes are study-defined TVB-N categories, not regulatory categories. MR, methyl red; BTB, bromothymol blue; BCP, bromocresol purple; BCG, bromocresol green.

This study established a controlled-laboratory prototype comprising four linked components: a KGM/gelatin/starch/SiO_2_ aerogel colorimetric array, standardized manual ROI extraction and fixed-order dye-grid construction, comparison of four lightweight CNN models with a compact feature-based multilayer perceptron, and a smartphone-oriented prototype workflow. In addition to the recorded fixed group-level split, four-fold specimen-group cross-validation was used to evaluate partition-related variability while keeping all storage-day observations and technical replicate images from each meat portion in the same data subset. The study did not evaluate independent purchasing batches, cross-device stability, automatic ROI localization or actual on-device performance. The overall sensing and classification workflow is illustrated in Figure 1.

## 2. Results and Discussion

### 2.1. Structural Characteristics and Dye Loading of the Aerogel Sensor

**Figure 2:**
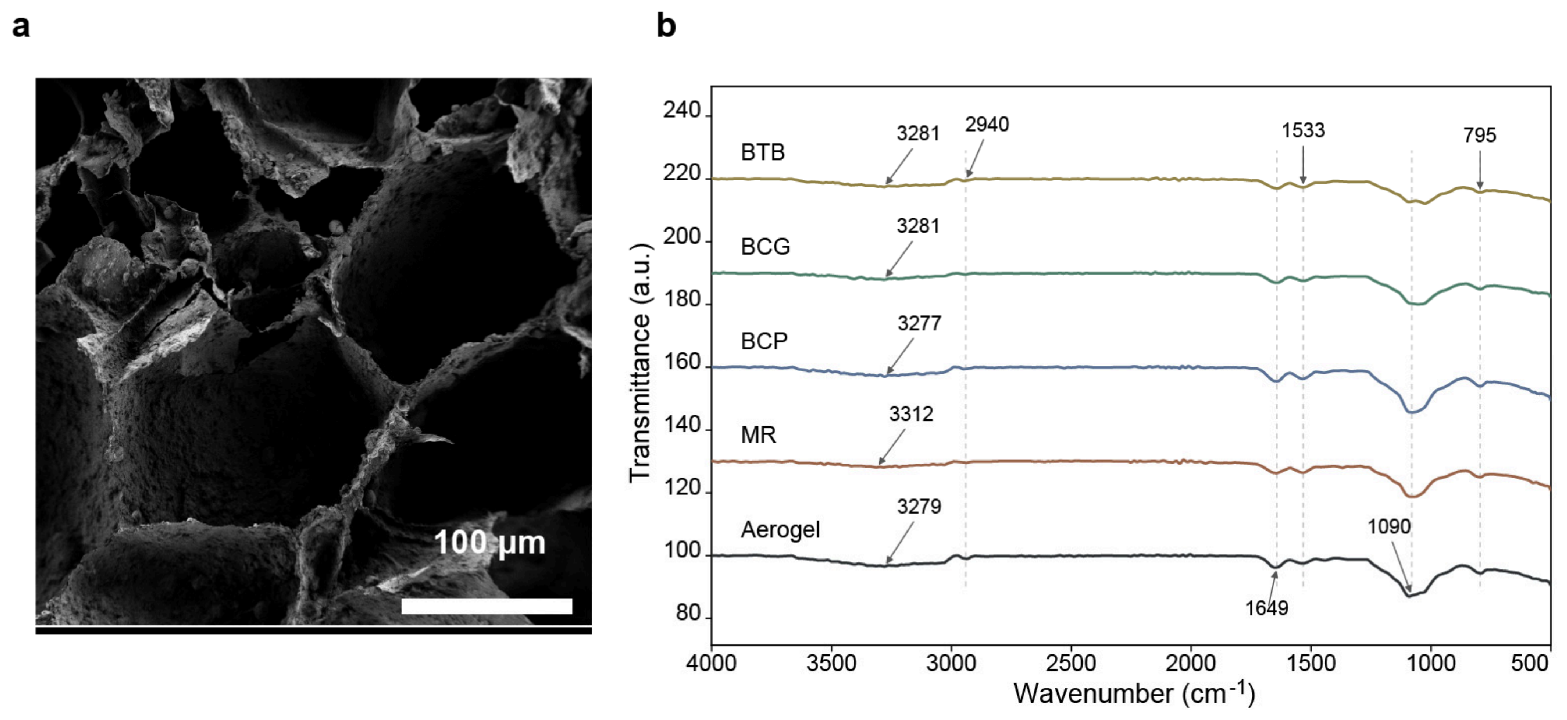
Porous microstructure and FT-IR characterization of the aerogel substrate. (**a**) Cross-sectional SEM image showing an interconnected pore structure; (**b**) Overlaid FT-IR spectra of the unloaded aerogel substrate and aerogels loaded with BTB, BCG, BCP and MR. BTB, BCG, BCP and MR denote aerogels loaded with the corresponding dyes, and Aerogel denotes the unloaded substrate. The spectra were vertically offset for clarity.

The scanning electron microscopy (SEM) and Fourier-transform infrared (FT-IR) characterization results are shown in Figure 2, and the detected absorption-band positions are listed in Supplementary Table S4. SEM images revealed a three-dimensional interconnected porous structure in the KGM/gelatin/starch/SiO_2_ composite aerogel, with open channels between adjacent pore walls. The SiO_2_ nanoparticles were employed to maintain the aerogel framework during freeze-drying and dye adsorption, based on our previous study [15]. To clarify the distribution of SiO_2_ nanoparticles within this framework, energy-dispersive X-ray spectroscopy (EDS) elemental mapping was performed (Supplementary Figure S12). The elemental maps showed broadly distributed Si and O signals throughout the examined aerogel region, supporting the incorporation and spatial distribution of the SiO_2_-containing phase within the composite matrix. Nanoparticle agglomeration was not quantitatively assessed. Furthermore, the porous properties of the aerogel were quantitatively evaluated using N_2_ adsorption–desorption isotherms. The aerogel exhibited a typical Type IV isotherm with a distinct hysteresis loop, which is characteristic of mesoporous structures (Supplementary Figure S10). BET analysis determined a specific surface area of 30.67 m^2^/g. The corresponding pore-size distribution curve showed that the pore diameters were mainly concentrated at ~50 nm (Supplementary Figure S11). These quantitative characteristics, together with the interconnected macroscopic pores observed via SEM, indicate a hierarchical porous architecture that could facilitate dye immobilization and the transport of volatile compounds within the sensing matrix. However, dye-loading capacity and gas-diffusion kinetics were not quantitatively evaluated in the present study. The FT-IR spectra of the unloaded and dye-loaded aerogels retained the principal bands of the substrate. The broad band at approximately 3277–3312 cm^−1^ was assigned mainly to O–H and N–H stretching vibrations, the band at approximately 2940 cm^−1^ to C–H stretching, and the bands near 1643 and 1533 cm^−1^ to the amide I and amide II vibrations of gelatin, respectively. Absorptions near 1080 and 795 cm^−1^ were associated mainly with polysaccharide C–O–C and Si–O–Si vibrations. After loading with MR, BCP, BCG or BTB, the overall spectral profiles and principal substrate bands were retained. Specifically, relative to the unloaded aerogel, the amide I and amide II minima shifted from 1649.1 and 1537.3 cm^−1^ to 1641.4–1645.3 and 1533.4–1535.3 cm^−1^, respectively. Differences were also observed in the broad O–H/N–H region and in the 1024.2–1080.1 cm^−1^ region (Supplementary Table S4). These spectra indicate that the bulk chemical structure of the aerogel remained largely unchanged after dye loading; they do not, by themselves, establish a specific binding mechanism.

### 2.2. Dynamic Color Response of the Sensor Array to NH_3_

**Figure 3:**
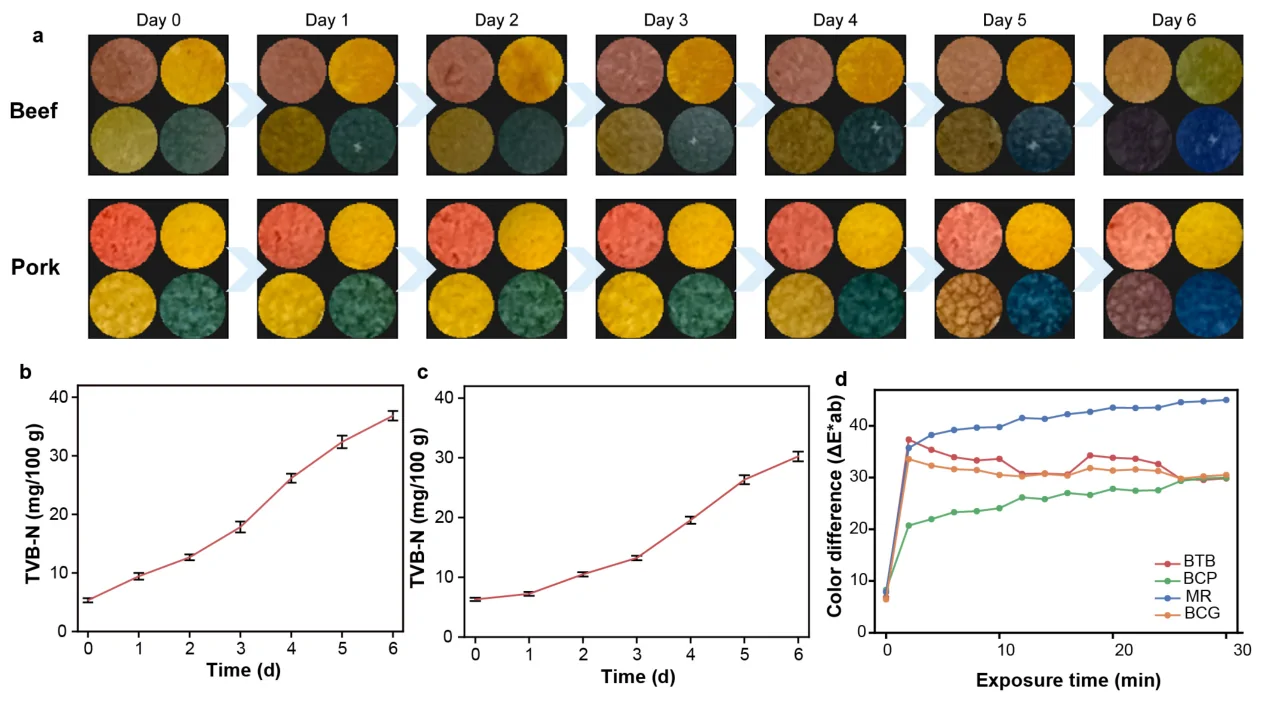
Changes in sensor-array color and TVB-N during chilled storage of beef and pork. (**a**) Sensor-array color-response sequences for beef and pork from day 0 to day 6; (**b**) Changes in beef TVB-N with storage time; (**c**) Changes in pork TVB-N with storage time; (**d**) ΔE*ab curves of the four dye units during continuous exposure to 10 ppm NH_3_. TVB-N values are mean ± standard deviation for three independent meat samples per meat type and storage day (n = 3).

Figure 3d shows the dynamic color responses of the four dye units during continuous exposure to 10 ppm NH_3_. All four units exhibited visible color changes, and most ΔE*ab values increased sharply within the first 2 min, demonstrating a rapid initial response under the tested exposure condition. NH_3_ was used as a representative volatile basic compound rather than as evidence that the array is ammonia-specific.

The response trajectories subsequently differed among the dyes. MR continued to increase and reached the largest ΔE*ab under the tested 10 ppm NH_3_ condition. BTB and BCG changed rapidly during the initial exposure and then approached plateaus, whereas BCP showed a slower, sustained increase. These dye-dependent trajectories formed a multidimensional color-response pattern; no separate experiment was conducted to attribute a change in classification performance to any individual dye trajectory. The intended sensing mode is a cross-reactive response to the combined basic volatile environment associated with spoilage rather than selective recognition of NH_3_ alone. Systematic cross-reactivity against individual spoilage volatiles was not evaluated.

### 2.3. TVB-N Changes and Freshness Grading During Beef and Pork Storage

The representative sensor responses and TVB-N changes are shown in Figure 3a–c. Sensor colors changed progressively from day 0 to day 6 (Figure 3a). Beef TVB-N increased from 5.33 ± 0.35 to 36.83 ± 0.81 mg/100 g, reaching the operational Sub-fresh stage on day 3 and the Spoiled stage from day 4 onward (Figure 3b). Pork TVB-N increased from 6.30 ± 0.26 to 30.23 ± 0.81 mg/100 g, reaching the operational Sub-fresh stage on day 4 and the Spoiled stage from day 5 onward (Figure 3c). TVB-N increased more rapidly in beef, while sensor color responses showed broadly similar temporal trends for both meat types. This comparison is descriptive because TVB-N was not measured for every imaged portion, and no portion-level correlation or calibration relationship was established.

### 2.4. Comparison of Lightweight-Model Classification Performance

**Figure 4:**
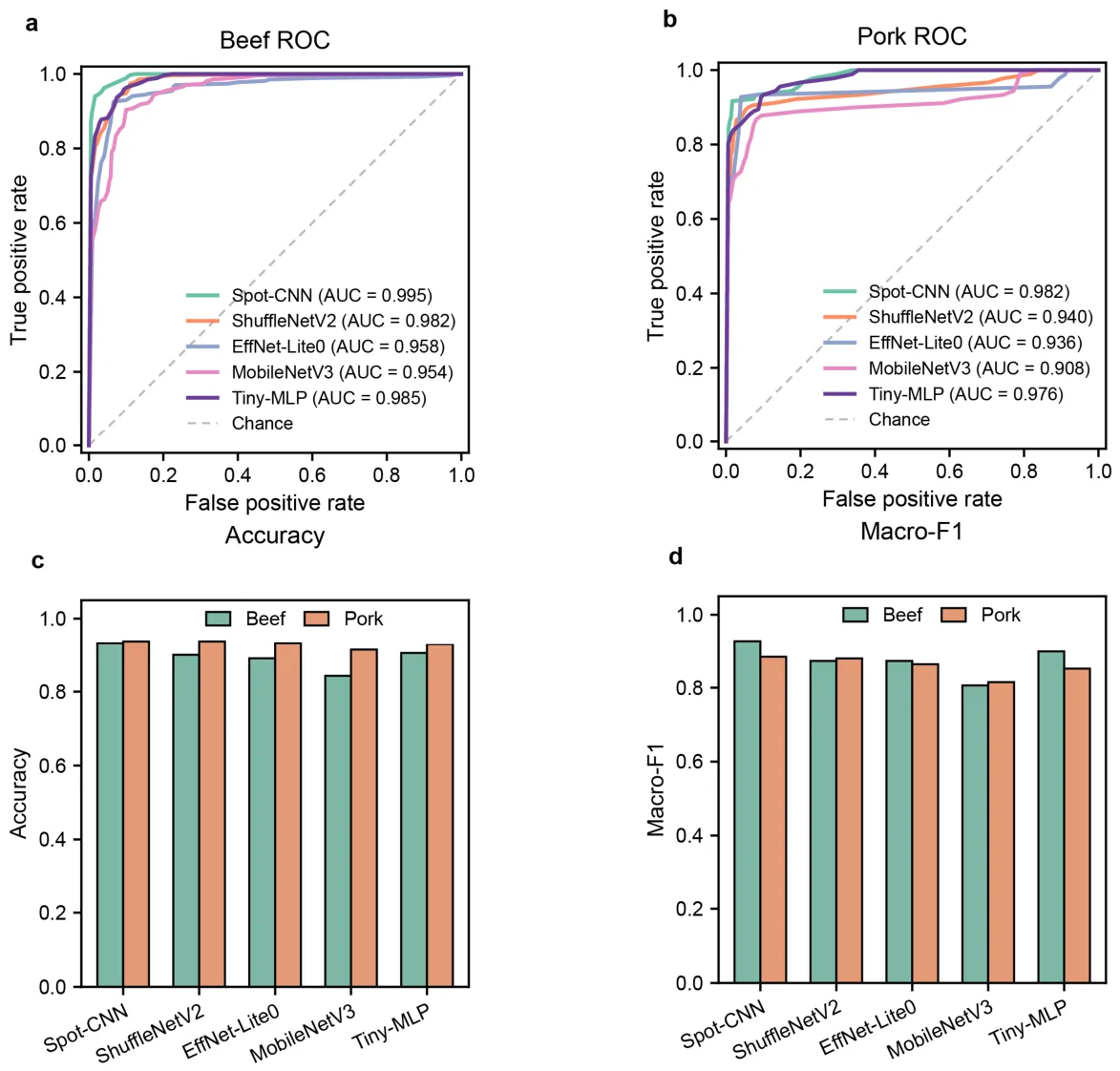
Overall model-performance comparison using image inputs or features derived from beef and pork 2 × 2 dye-grid images. (**a**) One-vs-rest macro-averaged ROC curves for the beef test set; (**b**) One-vs-rest macro-averaged ROC curves for the pork test set; (**c**) Accuracy of the five lightweight models on the beef and pork test sets; (**d**) macro-F1 of the five lightweight models on the beef and pork test sets. The ROC legends show the macro-AUC of each model, and the bar-chart values are taken from the current performance summary tables. ShuffleNetV2, EffNet-Lite0 and MobileNetV3 denote ShuffleNetV2-0.5x, EfficientNet-Lite0 and MobileNetV3-Small, respectively. These are image-unit-level results under the fixed group split; each test set contained 210 images from 10 independently stored meat portions.

Table 1 and Table 2 summarize image-unit-level classification performance and theoretical computational cost under the recorded fixed 20:10:10 group split. Each test set contained 210 dye-grid images derived from 10 independently stored meat portions; these images are repeated observations and do not represent 210 biologically independent samples. On the beef test set, Spot-CNN achieved the highest accuracy (0.933), macro-F1 (0.928) and macro-AUC (0.995). On the pork test set, Spot-CNN and ShuffleNetV2-0.5x achieved the same accuracy (0.938), while Spot-CNN yielded the higher macro-F1 (0.884) and macro-AUC (0.982). These fixed-split results identified Spot-CNN as the performance-prioritized model for that partition. The overall comparison is shown in Figure 4.

Tiny-MLP provided the lowest theoretical computational cost. It contained 0.013 M parameters and required <0.001 G floating-point operations (FLOPs), approximately 1/20 and 1/400 of the corresponding Spot-CNN values, respectively. Tiny-MLP achieved accuracies of 0.905 and 0.929 and macro-AUC values of 0.985 and 0.976 on the beef and pork test sets, respectively. These results show that a compact feature-based model retained substantial classification performance under the standardized ROI representation. Parameter count and FLOPs indicate deployment potential, but they do not measure latency or memory use on an actual mobile device.

Among the remaining convolutional models, ShuffleNetV2-0.5x matched the Spot-CNN accuracy on the pork dataset but had lower macro-F1 (0.879 vs. 0.884) and macro-AUC (0.940 vs. 0.982). EfficientNet-Lite0 and MobileNetV3-Small used more parameters than Tiny-MLP and did not exceed Spot-CNN in overall performance. Thus, under the recorded fixed partition, Spot-CNN offered the strongest performance profile, whereas Tiny-MLP offered the lowest theoretical computational cost.

To assess partition-related variability, four-fold specimen-group cross-validation was additionally performed using the separately stored meat portion as the grouping unit. Each rotation contained 20 training, 10 validation and 10 outer-test portions, corresponding to 420, 210 and 210 image units. All storage days and technical replicate images from each portion remained in one role. At the image-unit level, Tiny-MLP yielded the highest mean beef accuracy (93.21 ± 3.42%) and macro-F1 (0.9229 ± 0.0410), whereas Spot-CNN yielded the highest mean pork accuracy (95.12 ± 2.14%) and macro-F1 (0.9274 ± 0.0306). The corresponding macro-AUC values were 0.9884 ± 0.0071 and 0.9970 ± 0.0020. Values are fold-wise means ± sample standard deviations and describe partition variability rather than 95% confidence intervals. Full results are provided in Supplementary Figure S6 and Supplementary Table S9a,b.

To reduce the statistical weight assigned to the three technical replicate images acquired for each portion-day, an additional portion-day-level analysis was performed by averaging the predicted class probabilities of the three replicate images. Under the fixed test split, all three technical replicates yielded identical predicted classes in 74.3–95.7% of beef portion-day groups and 91.4–100.0% of pork groups across the five models. Probability aggregation reduced each fixed test set from 210 image units to 70 portion-day units. For Spot-CNN and Tiny-MLP, respectively, beef portion-day accuracy/macro-F1/macro-AUC were 0.9143/0.9134/0.9956 and 0.9143/0.9114/0.9858; the corresponding pork values were 0.9429/0.8942/0.9809 and 0.9286/0.8519/0.9766. Across the four outer-test folds, triplicate prediction consistency ranged from 80.0% to 97.1% for beef and from 86.1% to 98.6% for pork. At the portion-day level, Tiny-MLP achieved the highest mean accuracy and macro-F1 for beef (93.57 ± 3.78%; 0.9267 ± 0.0455) and pork (95.00 ± 2.47%; 0.9237 ± 0.0287), whereas Spot-CNN retained the highest pork macro-AUC (0.9973 ± 0.0021). Full technical-replicate consistency and portion-day-level results for all five models are provided in Supplementary Tables S12–S14.

The four-fold analysis also quantified performance for the underrepresented Sub-fresh class. Mean Sub-fresh F1-scores for Spot-CNN were 0.8233 ± 0.0736 for beef and 0.8548 ± 0.0495 for pork, whereas Tiny-MLP yielded 0.8891 ± 0.0667 and 0.8513 ± 0.0483, respectively. Results for all five models are provided in Supplementary Figure S8 and Supplementary Table S10a,b. Inverse-frequency weighting was used to address class-frequency imbalance during optimization but did not increase biological representation of the transition stage.

**Figure 5:**
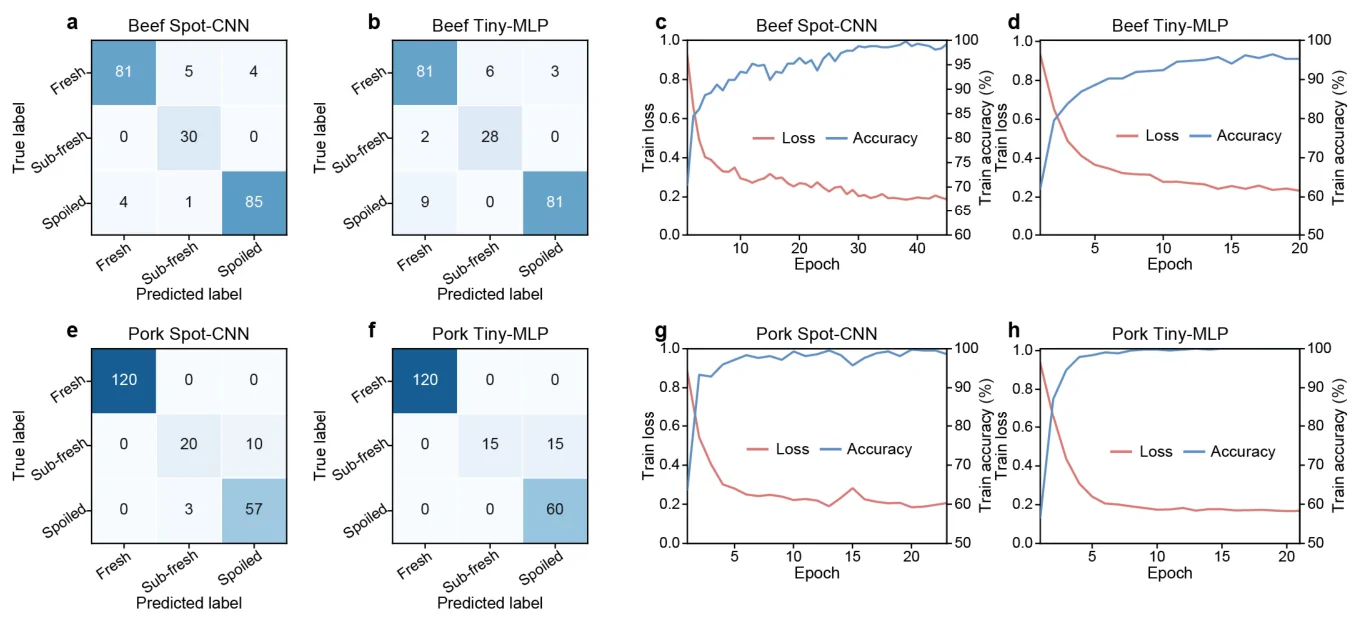
Confusion matrices and training processes of Spot-CNN and Tiny-MLP on the beef and pork datasets. (**a**,**b**,**e**,**f**), Test-set confusion matrices for beef Spot-CNN, beef Tiny-MLP, pork Spot-CNN and pork Tiny-MLP, respectively. Values in the matrices are raw dye-grid unit counts, and the classes are labeled Fresh, Sub-fresh and Spoiled; (**c**,**d**,**g**,**h**), Training-loss and training-accuracy curves for the corresponding models. These fixed-split metrics are calculated at the image-unit level; each test set contained 210 images from 10 independently stored meat portions.

The test-set confusion matrices in Figure 5a,b,e,f showed that error patterns depended on meat type and model. In the pork dataset, Sub-fresh image units were frequently classified as Spoiled, whereas all 30 Sub-fresh image units were correctly classified by Spot-CNN in the beef test set. This pattern is consistent with the comparatively small number of Sub-fresh samples and the fact that the operational classes represent adjacent stages along a continuous spoilage process. The training curves in Figure 5c,d,g,h show the convergence behavior of Spot-CNN and Tiny-MLP under the specified training settings; both models converged within the recorded training epochs. Additional training histories, comparator confusion matrices and class-specific precision-recall curves are provided in Supplementary Figures S2–S5. For Spot-CNN, fixed-split Sub-fresh precision, recall and F1-score were 0.833, 1.000 and 0.909 for beef and 0.870, 0.667 and 0.755 for pork, respectively (Supplementary Table S3a,b). Day-level labels may introduce uncertainty near adjacent operational freshness boundaries. Class weighting cannot replace additional independent sampling of the transition stage.

Previous studies have combined colorimetric sensor arrays with deep learning for rapid meat-freshness recognition. Portable colorimetric platforms and bioinspired algorithms have shown potential for on-site detection [27], and microelectromechanical-system (MEMS) gas-sensor arrays have been used to classify meat type and freshness status [28]. The present comparison extends this application-oriented evidence by evaluating predictive metrics and theoretical computational cost under common data splits.

### 2.5. Smartphone Prototype Workflow for Rapid Screening

The preceding experiments support the construction of a smartphone-oriented prototype workflow for standardized image acquisition and freshness classification. This workflow represents a controlled proof of concept rather than a validated mobile application.

**Figure 6:**
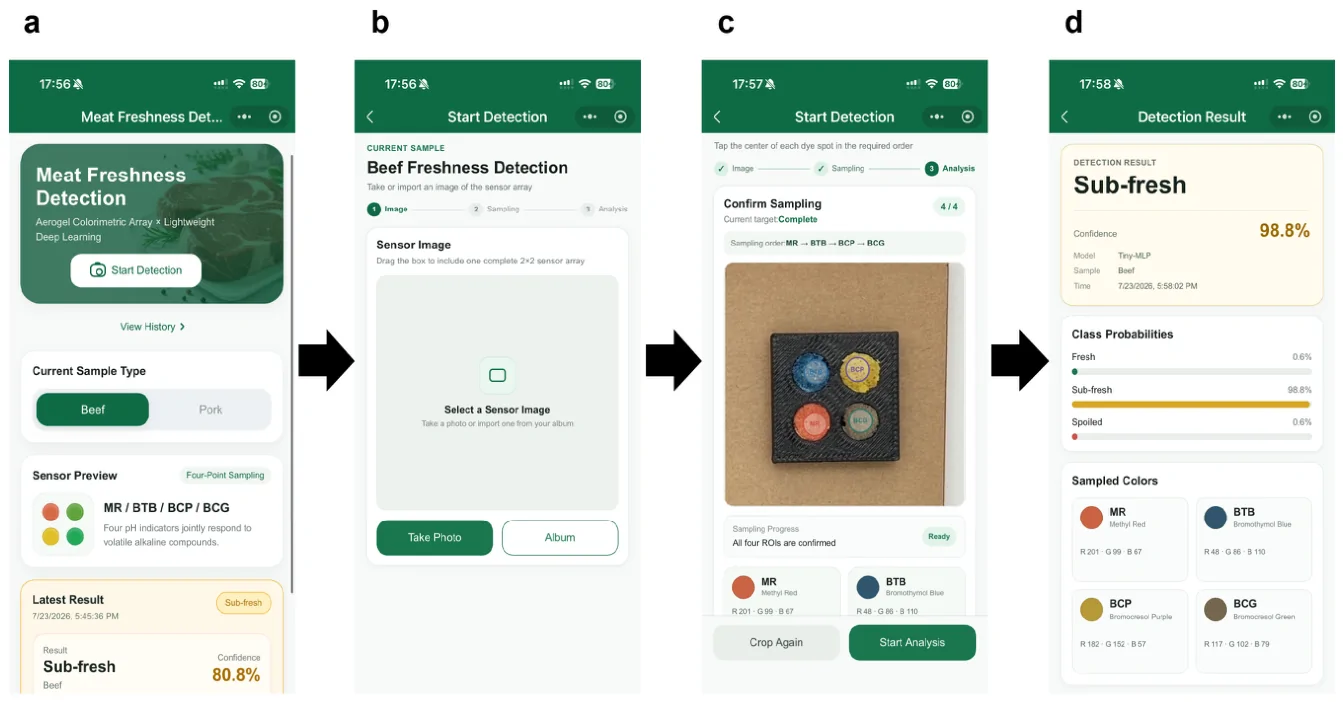
Prototype workflow for rapid smartphone-based meat freshness screening. (**a**) Selection of the meat type and initiation of detection; (**b**) capture or import of a sensor-array image; (**c**) manual confirmation and sampling of the four dye-responsive regions in the fixed order MR, BTB, BCP and BCG; (**d**) display of the predicted class, class probabilities and sampled colors. The workflow is a controlled proof of concept; device-level latency, memory consumption and operating stability were not evaluated.

The prototype workflow proceeded from image acquisition to freshness output (Figure 6). The sensor array was removed from the sealed storage container and immediately placed in the standardized image-acquisition box. A smartphone image was captured under constant artificial white light at a distance of approximately 15 cm. An operator then manually located and cropped the four dye-responsive regions in the fixed order MR, BTB, BCP and BCG. The regions were resized and concatenated into a 2 × 2 dye-grid image, from which the 78-dimensional feature vector was derived for Tiny-MLP; the CNN models used the dye-grid image directly. The lightweight model then generated probabilities for the Fresh, Sub-fresh and Spoiled classes.

Spot-CNN (0.250 M parameters) can be considered a performance-prioritized candidate when computational resources are relatively abundant, whereas Tiny-MLP (0.013 M parameters, <0.001 G FLOPs) represents a lower-cost candidate for further evaluation in resource-constrained settings. These metrics quantify theoretical model complexity only; they do not establish real-time operation or successful on-device deployment. The standardized ROI construction removed background pixels and retained the four sensing regions in a fixed arrangement. Whether this procedure improves cross-device consistency remains to be tested.

At the current prototype stage, the four dye-responsive regions are manually localized and cropped in the fixed order MR, BTB, BCP and BCG. Advancing the workflow toward a practical smartphone implementation will require automatic ROI localization and direct evaluation of latency, memory consumption and operational stability. Cross-device robustness must also be assessed using multiple smartphone models and lighting conditions. The prototype output is intended for rapid screening and risk indication and cannot replace standard measurements such as TVB-N.

### 2.6. Limitations and Future Work

This study has several scope limitations. Beef and pork were each obtained from one purchasing batch in one laboratory environment, without variation in supplier, animal source, packaging format or cold-chain history. High image-level AUC values therefore should not be interpreted as evidence of broad biological generalization. Although four-fold specimen-group cross-validation reduced dependence on one fixed partition, only 40 independently stored imaging portions were available per meat type. The additional portion-day aggregation reduced the statistical weight of the three technical replicate images, but observations collected across storage days from the same meat portion remained longitudinally repeated measurements rather than independent biological samples. The Sub-fresh stage was comparatively underrepresented and was assigned using day-level labels derived from TVB-N progression rather than a TVB-N measurement for every imaged portion, introducing potential label uncertainty near class boundaries.

Material, sensing and deployment limitations also remain. The four colorimetric dyes were used as laboratory sensing reagents, and the food-contact safety of the dye-loaded aerogel was not evaluated in the present study. Although the sensor array was mounted on the inner lid and physically separated from the meat, potential migration or unintended transfer of dye-related components to the food was not experimentally quantified. Before practical packaging applications, dedicated migration testing and toxicological and food-contact safety evaluation of the sensing materials will therefore be required. Nitrogen physisorption quantified the surface area and model-dependent pore sizes of the measured aerogel sample, but bulk porosity, gas diffusion and variability across independently prepared batches remain unquantified. NH_3_ exposure characterized the response to one representative basic volatile but did not establish analyte specificity or systematic cross-reactivity. The ΔE*ab and TVB-N results were compared descriptively because they were not paired for individual imaged portions. Images were acquired with one iPhone 16 Pro under fixed white light, ROIs were manually selected, and no on-device latency, memory or stability tests were performed. Future work should therefore include independent multi-batch sampling, increased transition-stage representation, further material characterization, multi-volatile cross-reactivity tests, automatic ROI localization and color-reference calibration across devices. Finally, comparison with additional quality indicators, including pH and TVC, could clarify how the sensor responses relate to complementary measures of meat spoilage.

## 3. Conclusions

This study establishes a controlled-laboratory prototype integrating a porous KGM/gelatin/starch/SiO_2_ aerogel colorimetric sensor array, standardized manual ROI image construction and lightweight-model comparison for beef and pork freshness assessment. Sensor ΔE*ab and TVB-N showed broadly similar temporal trends without establishing a portion-level quantitative correlation. Under the fixed group-level split, Spot-CNN achieved accuracies of 0.933 and 0.938 and macro-AUC values of 0.995 and 0.982 for beef and pork, respectively. In four-fold specimen-group cross-validation at the image-unit level, Tiny-MLP achieved the highest mean beef accuracy (93.21 ± 3.42%), whereas Spot-CNN achieved the highest mean pork accuracy (95.12 ± 2.14%). After aggregating the three technical-replicate probability vectors within each portion-day, Tiny-MLP achieved the highest mean portion-day accuracy for both beef (93.57 ± 3.78%) and pork (95.00 ± 2.47%), while Spot-CNN retained the highest pork portion-day macro-AUC (0.9973 ± 0.0021). Tiny-MLP contained 0.013 M parameters and required less than 0.001 G FLOPs.

The results support a smartphone-oriented proof-of-concept workflow under the tested laboratory conditions, not a fully automated or deployed diagnostic system. They do not establish generalization across purchasing batches, smartphone devices, illumination environments or distribution settings. The current workflow requires manual ROI localization, and no on-device latency, memory or stability tests were performed. Independent multi-batch validation, additional transition-stage samples, further aerogel characterization, cross-reactivity testing, automatic ROI localization and cross-device color standardization are required before practical deployment can be assessed.

## 4. Materials and Methods

### 4.1. Materials and Meat Sample Collection

The aerogel substrate was prepared from konjac glucomannan (KGM), gelatin (ref. 10010328; Sinopharm Chemical Reagent Co., Ltd., Shanghai, China) and potato starch, all of analytical grade. SiO_2_ nanoparticles (nominal particle size: 30 nm) were purchased from Macklin (Shanghai, China). SiO_2_ was included as an inorganic component intended to support the formation of the composite porous framework; however, no formulation control was used to isolate its individual contribution. Methyl red (MR), bromocresol purple (BCP), bromocresol green (BCG) and bromothymol blue (BTB) were used as pH-sensitive dyes. These dyes exhibit distinct acid-base response characteristics, and MR can produce a pronounced color response to volatile amines [29]. Combining multiple pH-sensitive dyes in an array enables cross-reactive responses that form differentiated color fingerprints [30]. Porphyrin and pH-indicator combinations have also been used to improve the stability of colorimetric arrays for pork-freshness monitoring [31].

Fresh beef and pork samples were purchased in one batch per meat type from a local supermarket on the day of the experiment. After transport to the laboratory, fascia and excess fat were removed. For each meat type, 40 portions of approximately 10 g were prepared for the longitudinal freshness-monitoring and sensor-imaging experiments. In addition, 21 separate meat portions per meat type were prepared exclusively for destructive TVB-N determination, corresponding to three independent portions at each of the seven storage time points. The additional TVB-N portions were stored under the same 4 °C conditions as the imaging portions. At each time point from day 0 to day 6, three TVB-N portions were removed for analysis and were not subsequently used for image acquisition. Thus, 61 meat portions were used per meat type, corresponding to 122 portions in the complete beef-and-pork experiment. Image acquisition and TVB-N measurements were performed at the same fixed time each day. Detailed dataset composition and experimental-unit grouping information are summarized in Supplementary Table S1a,b, and the TVB-N sampling design is clarified in Supplementary Note S6.

### 4.2. Preparation of the Aerogel Colorimetric Sensor Array

Gelatin (2 g) was dissolved in 100 mL of distilled water by stirring at 300 r/min and 60 °C for 30 min. KGM (1 g), potato starch (2 g) and SiO_2_ nanoparticles (3 g) were then added sequentially. The stirring speed was increased to 800 r/min, and mixing continued for 1 h to obtain a uniform dispersion. The mixture was transferred into cylindrical molds (Φ3.5 cm × 1.8 cm) and aged at 4 °C for 1 h. It was then pre-frozen at −15 °C for 12 h and freeze-dried at −55 °C and 10 Pa for 48 h. The resulting aerogel disks were equilibrated at 25 °C and 55% relative humidity (RH) for 48 h before use.

**Figure 7:**
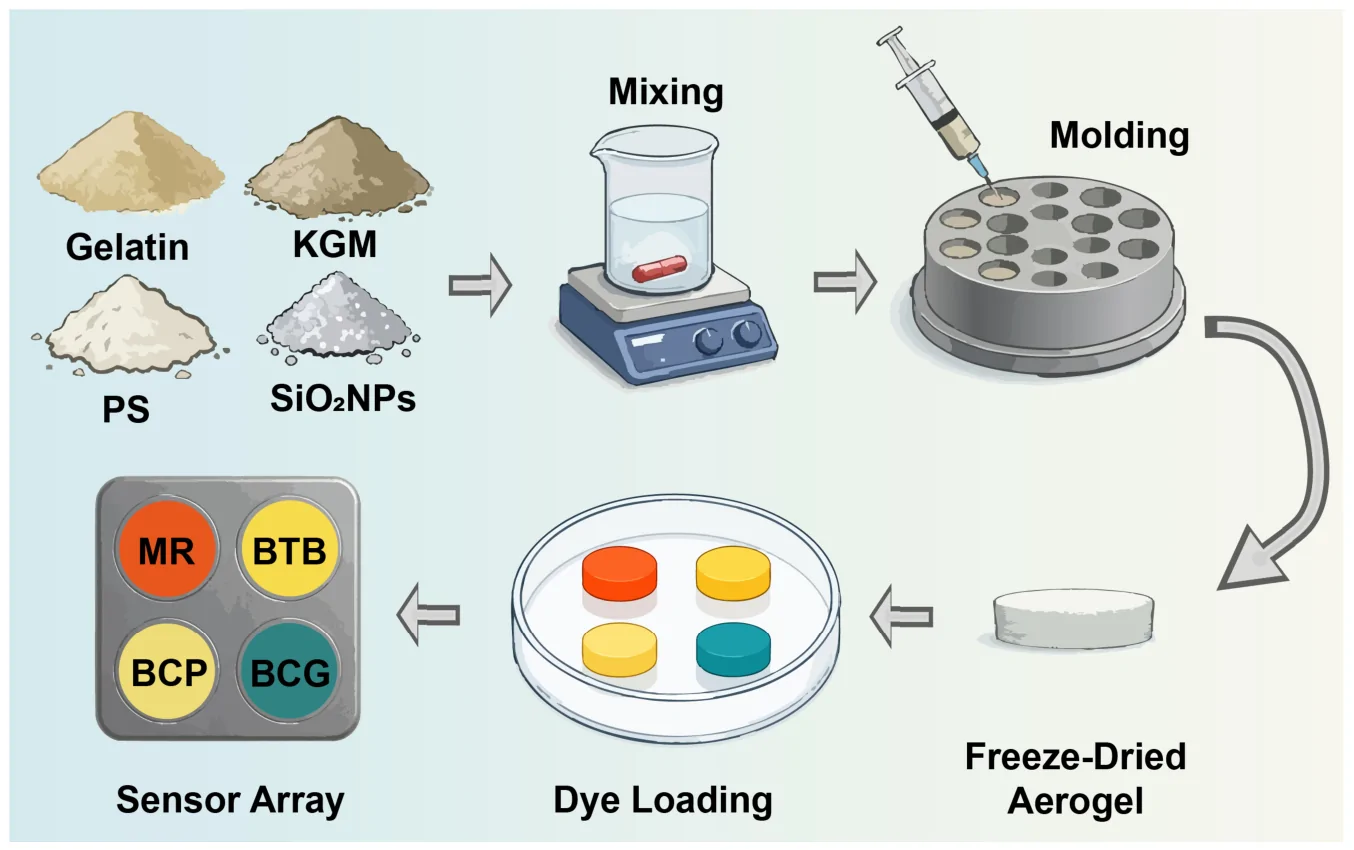
Preparation of the aerogel colorimetric sensor array. KGM, gelatin, potato starch and SiO_2_ nanoparticles were mixed, molded and freeze-dried to form a porous aerogel substrate. The substrate was then loaded separately with four pH-sensitive dyes: MR, BCP, BCG and BTB. The sensing units were assembled at fixed positions into a 2 × 2 array.

MR, BCP, BCG and BTB were separately dissolved in absolute ethanol to prepare 100 mg/L dye solutions. Aerogel disks were immersed in the corresponding solutions under stirring at 25 °C until uniform coloration was achieved, and then dried at 25 °C for 12 h to obtain single-dye sensing units. Four sensing units were assembled into a 2 × 2 aerogel colorimetric sensor array and equilibrated at 25 °C and 55% RH for 48 h before use. The preparation procedure of the aerogel colorimetric sensor array is summarized in Figure 7, and detailed sensor-fabrication conditions are listed in Supplementary Table S6.

### 4.3. Morphological and Structural Characterization of the Aerogel Sensor

Aerogel morphology was examined by scanning electron microscopy (SEM). Samples were cut to an appropriate size, and selected specimens were cryofractured in liquid nitrogen to expose the cross-section. The samples were mounted on stubs with conductive adhesive and sputter-coated with platinum under argon. Surface and cross-sectional micrographs were acquired using a JSM-6390LV scanning electron microscope (JEOL Ltd., Tokyo, Japan). Detailed characterization conditions are provided in Supplementary Table S8. Elemental mapping of Si, O and C in the KGM/gelatin/starch/SiO_2_ composite aerogel was performed using SEM coupled with energy-dispersive X-ray spectroscopy (EDS); the resulting maps are provided in Supplementary Figure S12.

The unloaded aerogel substrate and the sensing units loaded with MR, BCP, BCG and BTB were characterized using a Fourier-transform infrared spectrometer (IRTracer-100, Shimadzu, Japan). Before measurement, samples were dried in a forced-air oven at 60 °C for 12 h. Spectra were collected in attenuated total reflectance (ATR) mode over 4000–450 cm^−1^ and recorded as transmittance. Additional spectral information is provided in Supplementary Figure S1 and Supplementary Table S4, while the characterization conditions are summarized in Supplementary Table S8.

Nitrogen adsorption–desorption measurements were carried out at 77.3 K using a BSD-660S analyzer. The reported sample mass after degassing was 0.5761 g, and the degassing temperature was 120 °C. The multipoint BET fit used 10 adsorption points over P/P0 = 0.0503–0.2738. Pore-size distributions and branch-specific mean pore diameters were obtained using BJH analysis of the adsorption and desorption branches. Additional measurement details are provided in Supplementary Note S5 and Supplementary Table S11.

### 4.4. Dynamic Response of the Sensor Array to NH_3_

NH_3_ was selected as one representative volatile basic compound for dynamic-response characterization; the array was not designed or evaluated as an NH_3_-specific sensor. The target atmosphere was generated by volatilizing an ammonia solution and applying gas-bag dilution to obtain a nominal concentration of 10 ppm. The sensor array was placed in a sealed reaction chamber and continuously exposed for 30 min. Images were acquired every 2 min to record the temporal color-change process. Systematic selectivity and cross-reactivity tests against individual spoilage volatiles were not performed. Additional exposure conditions are provided in Supplementary Table S7.

Images were acquired in a standardized imaging system under constant artificial white light at a fixed distance of 15 cm. The main camera of an iPhone 16 Pro (Apple Inc., Cupertino, CA, USA) was used at a resolution of 3024 × 4032 pixels. Exposure compensation was fixed at +0.3 EV, while the remaining parameters were controlled automatically by the operating system. At each time point, three consecutive images of the same sensor array were acquired as technical replicate images; they were not treated as independent sensor experiments.

### 4.5. Chilled Meat Storage and Image-Acquisition Experiments

For each meat type, 40 separately stored meat portions were treated as independent experimental units and maintained in sealed containers at 4 °C for 7 d. Each approximately 10 g portion was individually placed in a sealed container and paired with an individual 2 × 2 aerogel colorimetric sensor array fixed to the inner lid of its corresponding container so that the sensor and meat shared the same headspace. The sensor array was physically separated from the meat and did not directly contact the food during storage. Sensor images were acquired every 24 h at seven time points from day 0 to day 6. At each time point, three consecutive images of each portion-associated sensor array were collected as technical replicate images, yielding 840 dye-grid image units for each meat type. For imaging, each array was removed from its container and immediately placed in the standardized image-acquisition box. After image acquisition, the array was returned to the corresponding container for continued storage at 4 °C. The smartphone was positioned approximately 15 cm directly above the sensor to maintain a consistent imaging distance and angle.

Images were acquired with the main camera of an iPhone 16 Pro (Apple Inc., Cupertino, CA, USA) at a resolution of 3024 × 4032 pixels. Exposure compensation was fixed at +0.3 EV, while the remaining parameters were controlled automatically by the operating system. The same lighting and acquisition settings were used for ROI extraction, color-response analysis and model construction.

### 4.6. TVB-N Measurement and Freshness-Label Definition

TVB-N was measured using the microdiffusion method. At each storage time point, three additional independently stored meat portions per meat type, separate from the 40 portions used for longitudinal sensor imaging, were destructively analyzed (n = 3) and were not subsequently used for image acquisition. Results are reported as mean ± standard deviation (mg/100 g). GB 2707–2016 specifies 15 mg/100 g as the relevant upper TVB-N limit for fresh livestock and poultry products [32]. Previous meat-freshness studies have used the 15–20 mg/100 g interval to represent an intermediate or less-fresh stage and values above 20 mg/100 g to indicate spoilage [27]. Accordingly, for model development in the present study, TVB-N ≤ 15 mg/100 g, 15 mg/100 g < TVB-N ≤ 20 mg/100 g and TVB-N > 20 mg/100 g were operationally defined as Fresh, Sub-fresh and Spoiled, respectively. The 20 mg/100 g boundary was therefore used as a literature-supported operational model-development threshold rather than as a universal regulatory limit. Detailed TVB-N values are provided in Supplementary Table S5.

Accordingly, beef observations from days 0–2, day 3 and days 4–6 were labeled Fresh, Sub-fresh and Spoiled, respectively; pork observations from days 0–3, day 4 and days 5–6 received the corresponding labels. All sensor images from the same meat type and storage day received the same day-level label because TVB-N was not measured separately for every imaged portion. This strategy assumes limited within-day biological variation and may introduce label uncertainty near the Fresh/Sub-fresh and Sub-fresh/Spoiled boundaries.

### 4.7. Sensor-Array Image Preprocessing and Sample Construction

After image acquisition, the four dye-responsive regions in each image were annotated as polygons using LabelMe [33]. The ROIs were cropped in the fixed order MR, BTB, BCP and BCG. Each ROI was resized to 64 × 64 pixels using bilinear interpolation, and pixels outside the annotated polygon were assigned a constant white background. The four ROI patches were concatenated into a 128 × 128-pixel 2 × 2 dye-grid image. This procedure excluded the meat and imaging background from the model input and retained the four sensing regions in a standardized arrangement. Detailed image-processing and model input parameters are summarized in Supplementary Table S2.

The same dye-grid construction procedure was applied to the beef and pork datasets. For the CNN models, pixel intensities were scaled to [0, 1] and then normalized using channel-wise means of 0.485, 0.456 and 0.406 and standard deviations of 0.229, 0.224 and 0.225. During training, mild color jitter was applied with brightness, contrast and saturation factors of 0.025, 0.025 and 0.015, respectively. No image augmentation was applied to the validation or test sets.

### 4.8. Dataset Splitting and Leakage-Prevention Strategy

Beef and pork followed the same experimental-unit grouping strategy for dataset construction. For each meat dataset, 40 portion-associated sensing units were divided into training, validation and test subsets at a ratio of 20:10:10. All storage-day images and technical replicate images derived from the same experimental unit were assigned to the same subset to prevent data leakage. Thus, each separately stored meat portion and its associated sensor-array images were retained as one grouping unit throughout data partitioning.

The same image-processing and model-training procedures were applied to both datasets. Differences in class distributions originated only from the freshness-label assignment according to the TVB-N progression of each meat type. Detailed dataset distributions are provided in Supplementary Table S1a,b.

Model parameters were optimized using only the training set. The validation set was used for early-stopping decisions and model selection, whereas the test set was reserved for final performance evaluation. Because the class distribution was imbalanced, validation macro-F1 was used as the common model-selection metric. Additional training configurations are listed in Supplementary Table S2.

For four-fold specimen-group cross-validation, StratifiedGroupKFold was applied with random seed 42. In each rotation, one fold was used for outer testing, the next fold for validation and the remaining two folds for training, giving 20, 10 and 10 meat portions and 420, 210 and 210 image units, respectively. All storage days and technical replicate images from one meat portion remained in one role. Models were initialized independently in each fold, validation macro-F1 was used for checkpoint selection, and the outer test set was used only for final evaluation.

### 4.9. Training of Lightweight Classification Models

Guided by previous studies of food-sensor images [34], five lightweight models were compared. Spot-CNN, ShuffleNetV2-0.5x, EfficientNet-Lite0 and MobileNetV3-Small were convolutional neural network models receiving 128 × 128-pixel RGB dye-grid images. Spot-CNN was designed for the small 2 × 2 dye-grid inputs and comprised convolutional, pooling and fully connected classification components. Tiny-MLP was a compact feature-based multilayer perceptron using a 78-dimensional vector. For each ROI, means and standard deviations from the RGB, hue-saturation-value (HSV) and CIE L*a*b* (Lab) channels yielded 18 features, giving 72 features across four ROIs. Six pairwise Euclidean distances between mean Lab vectors produced the remaining features. Tiny-MLP followed a 78→96→48→3 architecture with rectified linear unit (ReLU) activation, batch normalization after the first hidden layer, dropout rates of 0.20 and 0.10, and a three-unit output layer.

Within each meat dataset, all models used the same image-processing, optimization and model-selection procedures for a given partition. Training used cross-entropy loss and the AdamW optimizer with inverse-frequency class weighting. The same procedures were retained for four-fold specimen-group cross-validation, and class weights were calculated from the corresponding training subset only.

The learning rate, batch size, weight decay, label smoothing, maximum number of training epochs, patience, random seed and model-selection metric are reported in Supplementary Table S2. The verified computational environment used for the analysis included Python 3.10.20, PyTorch 2.11.0+cu128, torchvision 0.26.0+cu128, timm 1.0.27, scikit-learn 1.7.2, NumPy 1.26.4, pandas 2.3.3, Pillow 12.2.0, SciPy 1.15.3 and matplotlib 3.10.8; the environment is summarized in Supplementary Note S8.

### 4.10. Evaluation Metrics and Data Analysis

Classification performance was evaluated using accuracy, macro-precision, macro-recall, macro-F1 and macro-AUC. Macro-AUC was calculated by one-vs-rest binarization of the three classes followed by macro-averaging of the class-wise areas under the ROC curve. Confusion matrices were used to examine class-specific recognition, and one-vs-rest ROC curves were used to compare discrimination among the three freshness classes. The primary reported fixed-split and four-fold metrics were calculated at the dye-grid image-unit level. Each fixed test set contained 210 image units derived from 10 experimental-unit groups (10 portions × 7 storage days × 3 technical replicate images) and therefore did not represent 210 independent meat samples. To address the statistical weighting of the three technical replicate images, an additional portion-day-level analysis was performed. For each meat portion and storage day, the three class-probability vectors were averaged and the final class was assigned from the maximum averaged probability, yielding 70 portion-day units per fixed test set and per outer-test fold (10 portions × 7 storage days). Technical-replicate prediction consistency was quantified as the proportion of portion-day groups in which all three image-level predicted classes were identical. Portion-day observations collected across different storage days from the same meat portion remained longitudinally repeated measurements and were not treated as independent biological samples. Four-fold specimen-group results are reported as fold-wise mean ± sample standard deviation to describe partition variability, not as 95% confidence intervals.

Computational cost was summarized using parameter count (M) and floating-point operations (FLOPs, G). These metrics were used to compare theoretical computational requirements and deployment potential, rather than to represent measured on-device latency, memory consumption or energy use. Color responses were quantified using CIE ΔE*ab to describe color drift in each dye unit from day 0 to day 6 and were descriptively compared with the temporal trend in TVB-N. Additional model comparison results are provided in Supplementary Table S3a,b. A quantitative correlation was not calculated because TVB-N and image-derived color responses were not paired for each imaged meat portion. Cross-validation results are provided in Supplementary Tables S9a,b and S10a,b, and the technical-replicate consistency and portion-day-level analyses are provided in Supplementary Tables S12–S14.

## Figures and Tables

**Table 1:** Image-unit-level performance and computational cost of lightweight models for beef freshness classification under the fixed group split.

Model	Accuracy	Macro-Precision	Macro-Recall	Macro-F1	Macro-AUC	Params (M)	FLOPs (G)
Spot-CNN	0.933	0.914	0.948	0.928	0.995	0.250	0.407
ShuffleNetV2-0.5x	0.900	0.865	0.885	0.873	0.982	0.345	0.026
EfficientNet-Lite0	0.890	0.859	0.900	0.873	0.958	3.375	0.251
MobileNetV3-Small	0.843	0.802	0.819	0.806	0.954	1.521	0.038
Tiny-MLP	0.905	0.889	0.911	0.899	0.985	0.013	<0.001

**Table 2:** Image-unit-level performance and computational cost of lightweight models for pork freshness classification under the fixed group split.

Model	Accuracy	Macro-Precision	Macro-Recall	Macro-F1	Macro-AUC	Params (M)	FLOPs (G)
Spot-CNN	0.938	0.907	0.872	0.884	0.982	0.250	0.407
ShuffleNetV2-0.5x	0.938	0.927	0.861	0.879	0.940	0.345	0.026
EfficientNet-Lite0	0.933	0.937	0.844	0.864	0.936	3.375	0.251
MobileNetV3-Small	0.914	0.924	0.800	0.815	0.908	1.521	0.038
Tiny-MLP	0.929	0.933	0.833	0.852	0.976	0.013	<0.001

## Data Availability

Processed model-input data, code, model configurations, prediction probabilities and analysis scripts are publicly available at https://github.com/chenhanzhi605-eng/smartphone-aerogel-meat-freshness/releases/tag/v1.0.1 (accessed on 1 October 2026). Raw smartphone photographs, LabelMe files, allocation manifests and model weights are not included. Additional data are available from the corresponding author upon reasonable request.

## Supplementary Materials

The following supporting information can be downloaded at: https://www.mdpi.com/article/10.3390/gels12100910/s1: Supplementary Notes S1–S8; Figure S1: Unshifted FT-IR spectra of the unloaded aerogel and aerogels loaded with MR, BCP, BCG and BTB; Tables S1a–S8: supplementary information on dataset construction, model training, class-specific performance, FT-IR absorption bands, TVB-N measurements, sensor fabrication, NH_3_-response conditions and aerogel characterization; Figures S2–S5: training and validation curves, confusion matrices and class-specific precision-recall curves; Supplementary Note S4, Tables S9a,b, S10a,b, and Figures S6–S9: four-fold specimen-group cross-validation protocol and results; Supplementary Note S5 and Table S11: nitrogen-physisorption conditions and textural parameters; Supplementary Figures S10–S12: N_2_ adsorption–desorption isotherms, pore-size distribution and SEM–EDS elemental mapping, respectively; Supplementary Note S6: TVB-N sampling design clarification; Supplementary Note S7 and Tables S12–S14: technical-replicate consistency and portion-day-level analysis; Supplementary Note S8: verified software environment.
