# Supplementary material for: Smartphone-Assisted Aerogel Colorimetric Sensor Array Coupled with Lightweight Deep Learning for Multi-Meat Freshness Monitoring
Source: Gels. 2026 Oct 9;12(10):910. doi: 10.3390/gels12100910 (PMC13704372; doi:10.3390/gels12100910)
Supplement: Supplementary file 1 [file gels-12-00910-s001.zip › gels-4573513-supplementary.pdf]

Smartphone-assisted aerogel colorimetric sensor array coupled with lightweight deep learning for multi-meat freshness monitoring

Supplementary Notes

S1. Dataset hierarchy and group-level splitting

For each meat type, 40 separately stored meat portions were treated as independent experimental units at 4 °C for 7 d, and each portion was paired with an individual sensor array. Three consecutive images collected from one portion-associated sensor at one daily sampling point were treated as technical replicates. Each portion therefore yielded 21 dye-grid image units, giving 840 image units per meat type. All storage days and technical replicates associated with one portion remained in the same training, validation or outer-test role to prevent data leakage. These 40 portions constituted the longitudinal imaging dataset; the additional portions used exclusively for destructive TVB-N determination are described in Supplementary Note S6 and Supplementary Table S5.

S2. ROI processing and dye-grid construction

Four dye-responsive ROIs were extracted in the fixed order MR, BTB, BCP and BCG. The CNN models received 128×128-pixel RGB dye-grid images, whereas Tiny-MLP used a 78-dimensional feature vector comprising 72 ROI color-summary features and six pairwise Lab-distance features.

S3. FT-IR data handling

FT-IR spectra were acquired in attenuated total reflectance mode using an IRTracer-100 spectrometer (Shimadzu, Japan) over 4000–450 cm^{-1}; each exported spectrum comprised 1,842 points spanning 4000.36–449.41 cm^{-1}. Supplementary Figure S1 and Supplementary Table S4 use the original transmittance data without smoothing, normalization or peak-position alteration. Local transmittance minima were reported as absorption-band positions, and only vertical offsets were applied to the main-text stacked plot.

S4. Four-fold specimen-group cross-validation

Four-fold specimen-group cross-validation was performed separately for beef and pork using StratifiedGroupKFold with random seed 42. Each rotation used 20 meat portions for training, 10 for validation and 10 for outer testing, corresponding to 420, 210 and 210 image units. All storage-day observations and technical replicate images from one portion remained in one role. Models were independently initialized in each fold. Class weights were calculated from the corresponding training subset, validation macro-F1 was used for checkpoint selection, and the outer test set was used only for final evaluation. Fold summaries are reported as mean ± sample standard deviation and should not be interpreted as 95% confidence intervals.

S5. Nitrogen physisorption characterization

Nitrogen adsorption–desorption measurements were carried out at 77.3 K using a BSD-660S analyzer. The reported sample mass after degassing was 0.5761 g, and the degassing temperature was 120 °C. The multipoint BET fit used 10 adsorption points over P/P_0 = 0.0503–0.2738. Pore-size distributions and branch-specific mean pore diameters were obtained using BJH analysis of the adsorption and desorption branches. The instrument report lists one measured sample; no replicate-based uncertainty is inferred. The reported fitting correlation coefficient is $r = 0.99995$ (corresponding to $R^2 \approx 0.99990$).

S6. TVB-N sampling design clarification

For each meat type, the 40 separately stored meat portions described in Supplementary Note S1 were used for longitudinal sensor imaging. In addition, 21 separate meat portions per meat type were prepared exclusively for destructive TVB-N determination, corresponding to three independent portions at each of seven storage days. These additional TVB-N portions were stored under the same 4 °C conditions as the imaging portions and were not subsequently used for image acquisition. Thus, 61 meat portions were used per meat type, corresponding to 122 portions in the complete beef-and-pork experiment.

S7. Portion-day aggregation and technical-replicate consistency

Technical-replicate consistency was evaluated by determining whether the three image-level predicted classes within each portion-day group were identical. For portion-day aggregation, the three class-probability vectors were averaged and the class with the maximum averaged probability was assigned. Each fixed test set and each outer-test fold therefore contained 70 portion-day units rather than 210 image units. Observations from different storage days of the same meat portion remain longitudinally repeated measurements and are not independent biological samples.

S8. Software environment

The verified computational environment used Python 3.10.20, PyTorch 2.11.0+cu128, torchvision 0.26.0+cu128, timm 1.0.27, scikit-learn 1.7.2, NumPy 1.26.4, pandas 2.3.3, Pillow 12.2.0, SciPy 1.15.3 and matplotlib 3.10.8. PyTorch reported CUDA runtime 12.8. OpenCV was not used in the verified preprocessing, training or evaluation workflow. A public reproducibility package (release v1.0.1) containing processed model-input data, computational code, model configurations, saved prediction probabilities and analysis scripts is available at https://github.com/chenhanzhi605-eng/smartphone-aerogel-meat-freshness.

Supplementary Figure S1

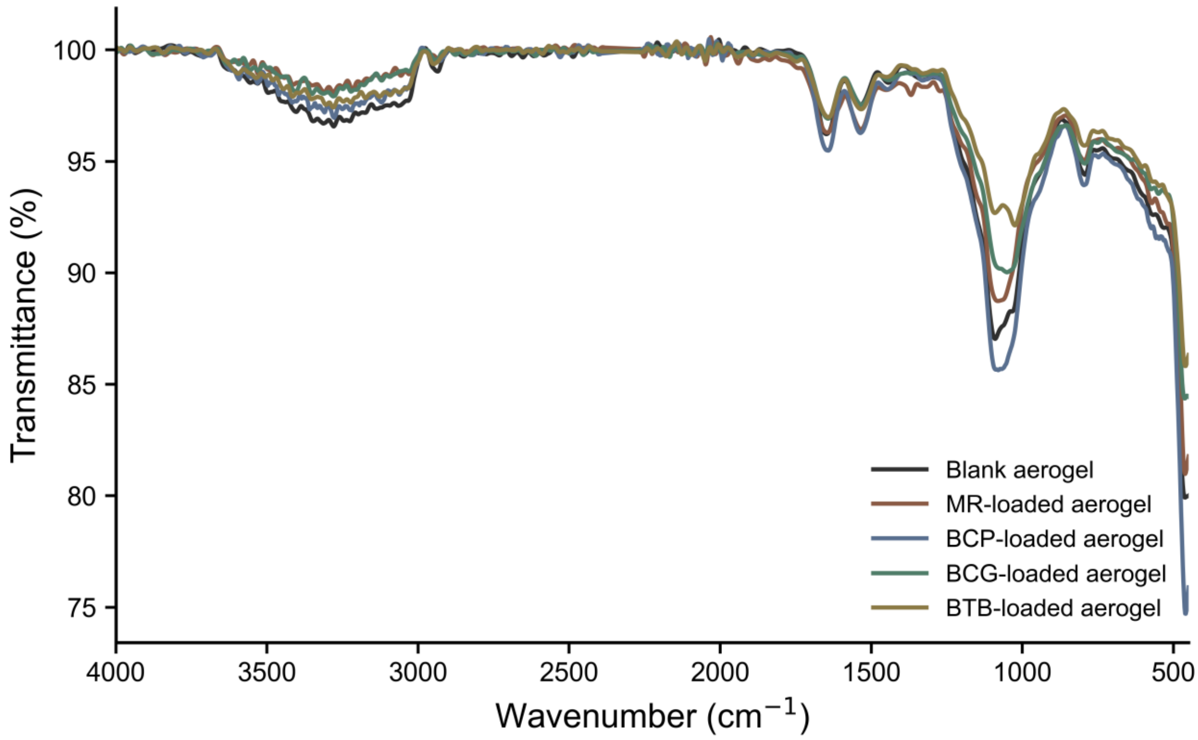

Supplementary Figure S1. Unshifted FT-IR spectra of the unloaded aerogel and aerogels loaded with MR, BCP, BCG and BTB. The curves show the original exported transmittance values without smoothing or normalization.

Supplementary Tables

Supplementary Table S1a. Dataset hierarchy and derivation of model-input units.

Dataset	Experimental units	Dye-grid image units	Grouping unit	Groups	Unit derivation
Beef	40	840	Separately stored meat portion	40	One four-ROI dye-grid per image unit
Pork	40	840	Separately stored meat portion	40	One four-ROI dye-grid per image unit

Three images acquired from the same imaging experimental unit at one sampling point were treated as technical replicates rather than independent experiments. The 40 experimental units listed in Supplementary Table S1a refer only to the longitudinal imaging dataset and exclude the 21 additional portions per meat type used exclusively for destructive TVB-N determination.

Supplementary Table S1b. Class distribution within the recorded group-level split.

Dataset	Split	Groups	Image units	Fresh	Sub-fresh	Spoiled	Dye-grid units
Beef	Train	20	420	180	60	180	420
Beef	Validation	10	210	90	30	90	210
Beef	Test	10	210	90	30	90	210
Pork	Train	20	420	240	60	120	420
Pork	Validation	10	210	120	30	60	210
Pork	Test	10	210	120	30	60	210

Fixed-split performance metrics were calculated at the dye-grid image-unit level. Each test set contained 210 image units derived from 10 independently stored meat portions and should not be interpreted as 210 biologically independent samples.

Supplementary Table S2. Training and input-processing parameters recorded in the active code and run configuration.

Parameter	Recorded value
ROI patch size	64×64 pixels
CNN dye-grid size	128×128 pixels
ROI crop scale	1.06 × annotation bounding-box side
ROI interpolation	Bilinear
Outside-ROI fill	Constant white (255, 255, 255)
Training color jitter	Brightness 0.025; contrast 0.025; saturation 0.015

Parameter	Recorded value
Validation/test augmentation	None
Loss	Cross-entropy
Class weighting	Inverse frequency
Label smoothing	0.03
Optimizer	AdamW
Initial learning rate	1×10^{-3}
Learning-rate scheduler	CosineAnnealingLR; T_max = 45
Batch size	32
Weight decay	1×10^{-3}
Maximum epochs	45
Early-stopping patience	9 epochs
Checkpoint selection	Validation macro-F1
Random seed	42
Deterministic algorithms	Enabled
Comparator initialization	No pretrained weights

Supplementary Table S3a. Class-specific test performance of the five models on the beef dataset.

Model	Class	Precision	Recall	F1-score	Support
Spot-CNN	Fresh	0.953	0.900	0.926	90
Spot-CNN	Sub-fresh	0.833	1.000	0.909	30
Spot-CNN	Spoiled	0.955	0.944	0.950	90
ShuffleNetV2-0.5x	Fresh	0.894	0.933	0.913	90
ShuffleNetV2-0.5x	Sub-fresh	0.714	0.833	0.769	30
ShuffleNetV2-0.5x	Spoiled	0.988	0.889	0.936	90
EfficientNet-Lite0	Fresh	0.891	0.911	0.901	90
EfficientNet-Lite0	Sub-fresh	0.700	0.933	0.800	30
EfficientNet-Lite0	Spoiled	0.987	0.856	0.917	90
MobileNetV3-Small	Fresh	0.835	0.900	0.866	90
MobileNetV3-Small	Sub-fresh	0.611	0.733	0.667	30
MobileNetV3-Small	Spoiled	0.961	0.822	0.886	90
Tiny-MLP	Fresh	0.880	0.900	0.890	90
Tiny-MLP	Sub-fresh	0.824	0.933	0.875	30
Tiny-MLP	Spoiled	0.964	0.900	0.931	90

Supplementary Table S3b. Class-specific test performance of the five models on the pork dataset.

Model	Class	Precision	Recall	F1-score	Support
Spot-CNN	Fresh	1.000	1.000	1.000	120
Spot-CNN	Sub-fresh	0.870	0.667	0.755	30
Spot-CNN	Spoiled	0.851	0.950	0.898	60
ShuffleNetV2-0.5x	Fresh	0.992	1.000	0.996	120
ShuffleNetV2-0.5x	Sub-fresh	0.947	0.600	0.735	30
ShuffleNetV2-0.5x	Spoiled	0.843	0.983	0.908	60
EfficientNet-Lite0	Fresh	1.000	1.000	1.000	120
EfficientNet-Lite0	Sub-fresh	1.000	0.533	0.696	30
EfficientNet-Lite0	Spoiled	0.811	1.000	0.896	60
MobileNetV3-Small	Fresh	0.984	1.000	0.992	120
MobileNetV3-Small	Sub-fresh	1.000	0.400	0.571	30
MobileNetV3-Small	Spoiled	0.789	1.000	0.882	60
Tiny-MLP	Fresh	1.000	1.000	1.000	120
Tiny-MLP	Sub-fresh	1.000	0.500	0.667	30
Tiny-MLP	Spoiled	0.800	1.000	0.889	60

Supplementary Table S4. Major absorption bands detected as local minima in the original FT-IR transmittance spectra.

Sample	Detected absorption-band positions (cm^{-1})
Unloaded aerogel	3279.0, 2935.7, 1649.1, 1537.3, 1089.8, 792.7
MR-loaded aerogel	3311.8, 2048.4, 1645.3, 1533.4, 1080.1, 794.7, 461.0
BCP-loaded aerogel	3277.1, 1643.4, 1535.3, 1080.1, 796.6, 459.1
BCG-loaded aerogel	3406.3, 3280.9, 1643.4, 1533.4, 1051.2, 792.7
BTB-loaded aerogel	3280.9, 2949.2, 2050.3, 1641.4, 1533.4, 1024.2, 794.7

Detected local minima are listed as source-data positions and are not, by themselves, evidence of a specific binding mechanism or new covalent-bond formation.

Supplementary Table S5. TVB-N source-data summary during chilled storage.

Storage day	Beef mean	Beef SD	Beef n	Pork mean	Pork SD	Pork n
0	5.33	0.35	3	6.30	0.26	3
1	9.43	0.58	3	7.23	0.32	3
2	12.67	0.50	3	10.50	0.36	3
3	17.83	0.93	3	13.23	0.38	3
4	26.17	0.76	3	19.57	0.60	3
5	32.37	1.08	3	26.33	0.76	3
6	36.83	0.81	3	30.23	0.81	3

TVB-N values are expressed as mg/100 g and were determined using three additional independently stored meat portions for each meat type at each storage day (n = 3). These portions were separate from the 40 longitudinal imaging portions, were destructively analyzed, and were not subsequently used for image acquisition.

Supplementary Table S6. Recorded sensor-fabrication conditions.

Parameter	Recorded value
Aerogel formulation	Gelatin 2 g; KGM 1 g; potato starch 2 g; SiO_2 nanoparticles 3 g; distilled water 100 mL
Initial dissolution	Gelatin dissolved at 300 r/min and 60 °C for 30 min
Composite mixing	KGM, potato starch and SiO_2 nanoparticles added sequentially; 800 r/min for 1 h
Molding and aging	Cylindrical molds (Φ3.5 cm × 1.8 cm); 4 °C for 1 h
Pre-freezing	−15 °C for 12 h
Freeze-drying	−55 °C and 10 Pa for 48 h
Dye solutions	MR, BCP, BCG and BTB separately prepared at 100 mg/L in absolute ethanol
Dye-loading method	Immersion adsorption until uniform coloration
Post-loading drying	25 °C for 12 h
Pre-use equilibration	25 °C and 55% relative humidity for 48 h
Array construction	Four sensing units assembled as a 2×2 array; model-input order MR, BTB, BCP and BCG
SiO_2 supplier	Macklin (Shanghai, China)
SiO_2 nominal particle size	30 nm

Supplementary Table S7. Recorded conditions for the dynamic NH_3-response experiment.

Parameter	Recorded value
Analyte	NH_3
Target concentration	10 ppm
Generation and dilution	Ammonia solution evaporation followed by gas-bag dilution
Exposure environment	Sealed reaction chamber
Exposure duration	30 min
Image interval	2 min
Image replicates	Three consecutive technical images from the same sensor array at each time point; not independent sensor experiments
Imaging device	iPhone 16 Pro main camera; 3024 × 4032 pixels; exposure compensation +0.3 EV
Imaging geometry	Standardized artificial white-light box; camera approximately 15 cm above the sensor

Supplementary Table S8. Recorded characterization conditions for the aerogel.

Parameter	Recorded value
SEM instrument	JSM-6390LV scanning electron microscope (JEOL Ltd., Japan)
SEM sample preparation	Liquid-nitrogen cryofracture; conductive adhesive; platinum coating under argon
FT-IR instrument	IRTracer-100 Fourier-transform infrared spectrometer (Shimadzu, Japan)
FT-IR mode	Attenuated total reflectance (ATR)
FT-IR range	4000–450 cm^{-1}
FT-IR sample pretreatment	Forced-air drying at 60 °C for 12 h
FT-IR output	Transmittance
Si elemental mapping/distribution characterization	SEM coupled with energy-dispersive X-ray spectroscopy (EDS); Si, O and C elemental maps
Nitrogen-physisorption instrument	BSD-660S
Adsorptive and analysis temperature	N_2 at 77.3 K
Post-degassing sample mass	0.5761 g
Degassing temperature	120 °C

Supplementary Figures S2–S5

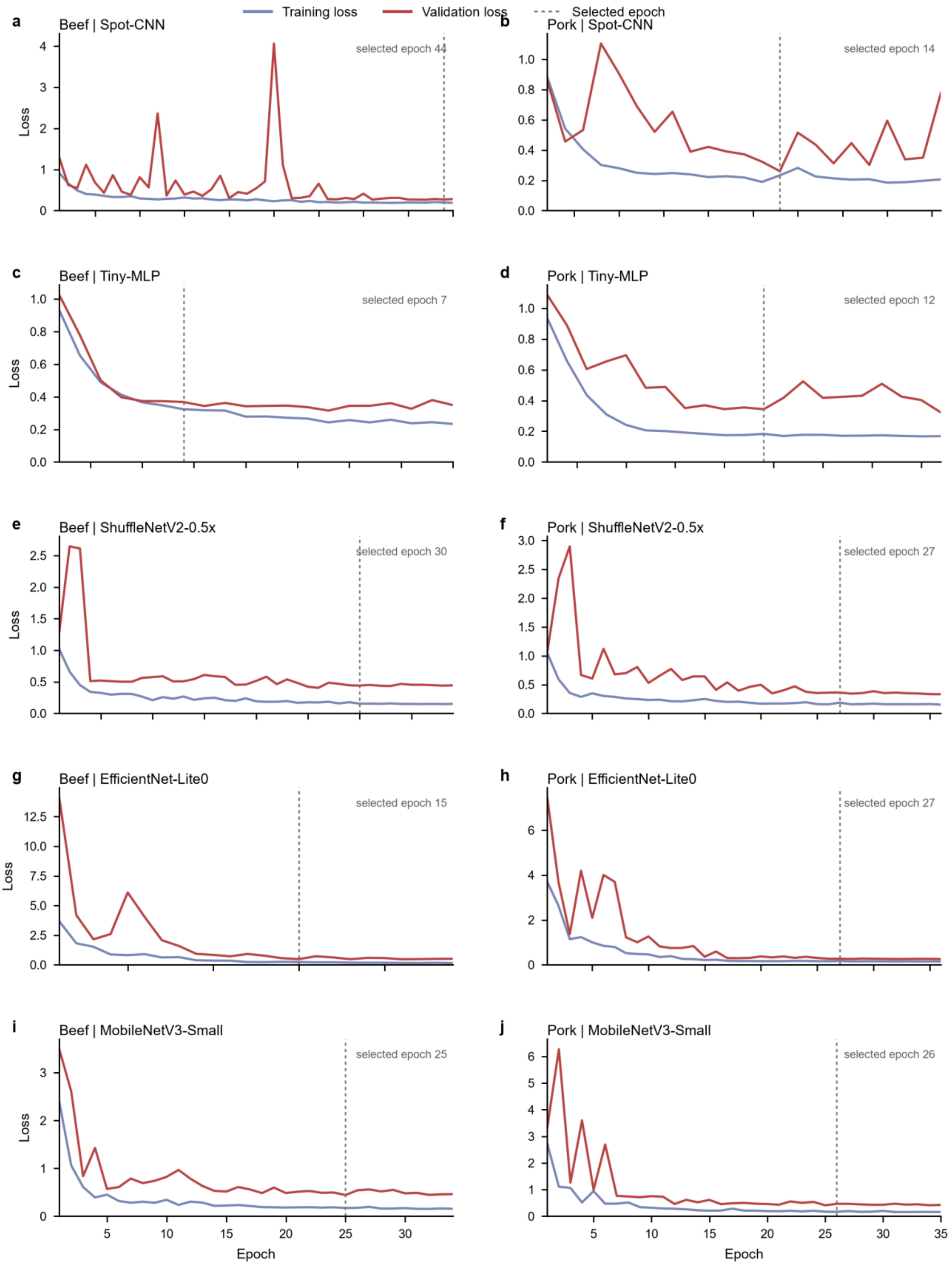

Supplementary Figure S2. Training and validation loss curves for the evaluated models. Curves were obtained from the recorded training histories without smoothing.

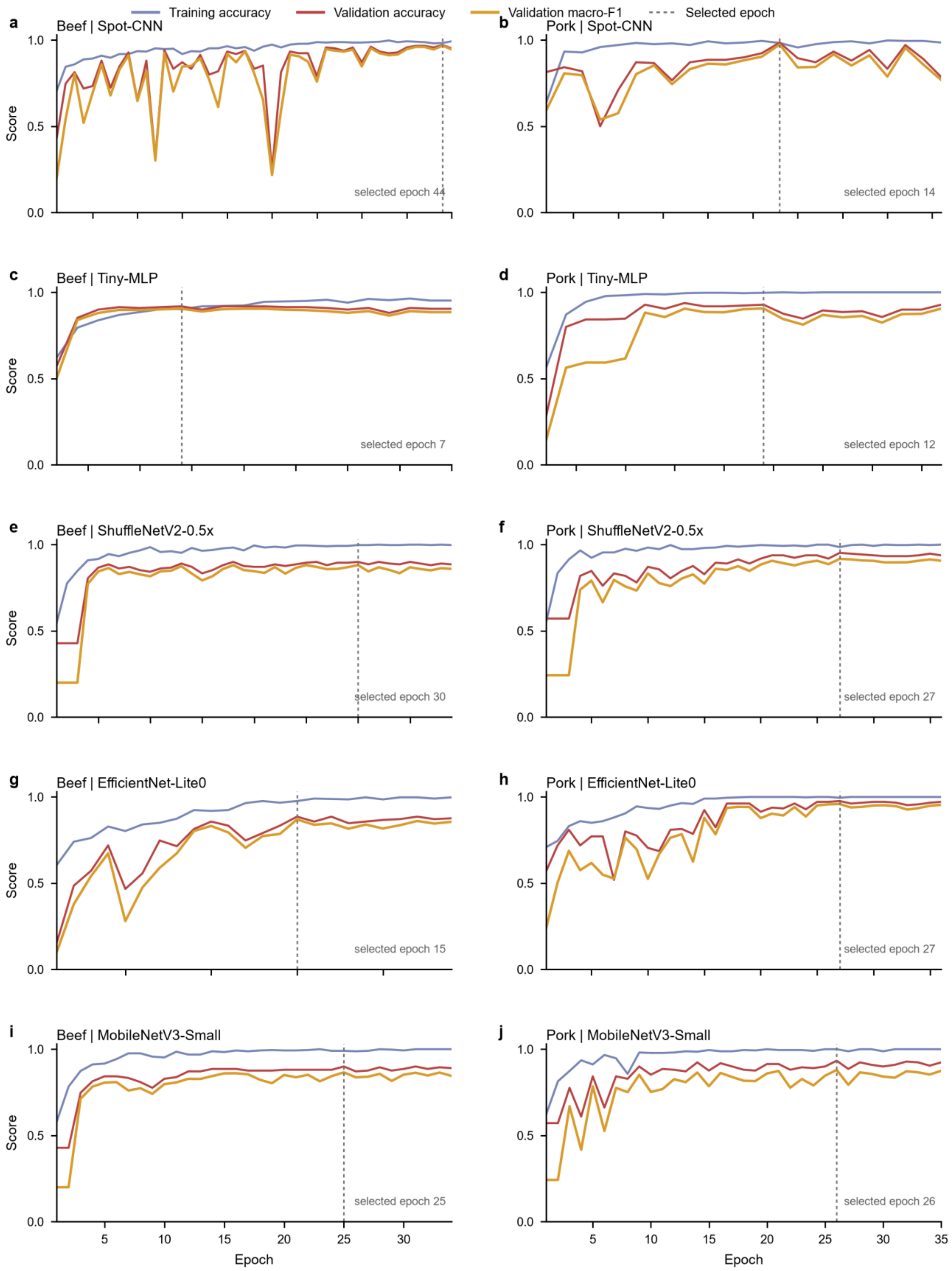

Supplementary Figure S3. Training and validation accuracy curves and validation macro-F1 trajectories for the evaluated models.

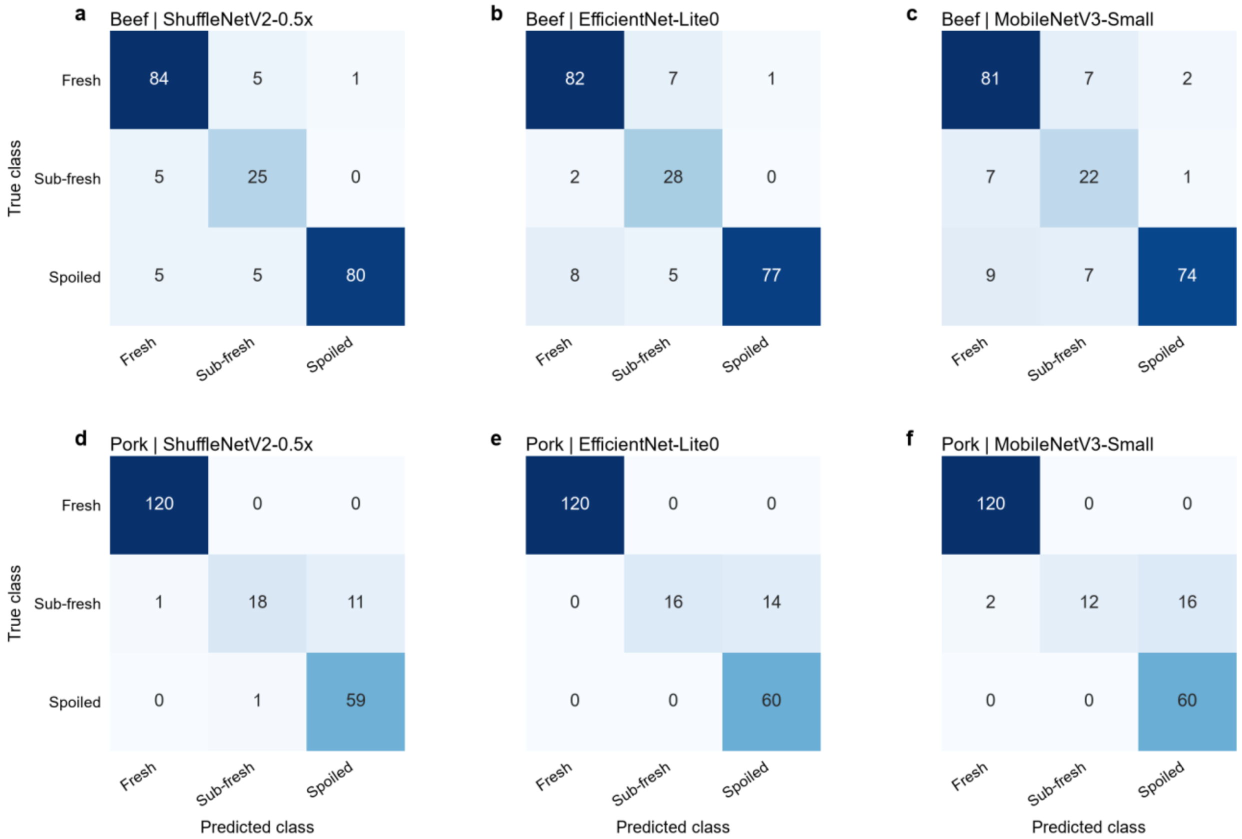

Supplementary Figure S4. Test-set confusion matrices for the three comparator convolutional networks. (a)–(c), ShuffleNetV2-0.5x, EfficientNet-Lite0 and MobileNetV3-Small on the beef test set, respectively; (d)–(f), the corresponding models on the pork test set. Cells show raw dye-grid unit counts (n = 210 per test set).

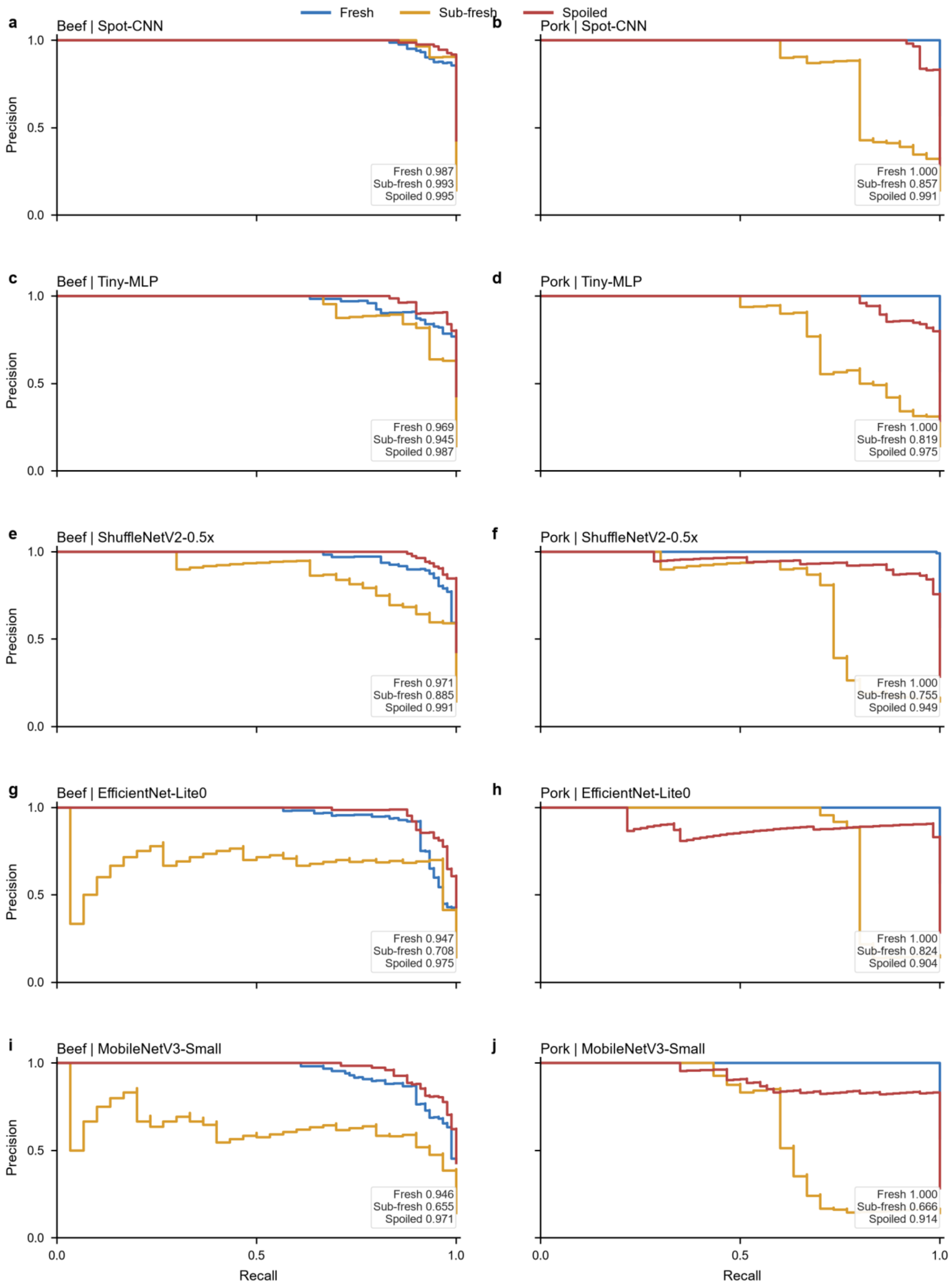

Supplementary Figure S5. Class-specific precision-recall curves for the evaluated models.

Supplementary cross-validation tables

Supplementary Table S9a. Beef overall performance across four specimen-group outer folds. Values are mean ± sample SD; accuracy is expressed as percent.

Model	Accuracy (%)	Macro- Precision	Macro- Recall	Macro-F1	Macro-AUC
Spot-CNN	91.79 ± 2.50	0.9073 ± 0.0182	0.8935 ± 0.0545	0.8965 ± 0.0367	0.9889 ± 0.0070
ShuffleNetV2-0.5x	86.79 ± 2.07	0.8354 ± 0.0182	0.8639 ± 0.0259	0.8456 ± 0.0202	0.9589 ± 0.0115
EfficientNet-Lite0	87.26 ± 2.14	0.8405 ± 0.0279	0.8620 ± 0.0235	0.8482 ± 0.0270	0.9588 ± 0.0134

Model	Accuracy (%)	Macro- Precision	Macro- Recall	Macro-F1	Macro-AUC
MobileNetV3-Small	82.86 ± 1.03	0.7971 ± 0.0148	0.8333 ± 0.0240	0.8076 ± 0.0180	0.9498 ± 0.0073
Tiny-MLP	93.21 ± 3.42	0.9150 ± 0.0371	0.9343 ± 0.0466	0.9229 ± 0.0410	0.9884 ± 0.0071

Supplementary Table S9b. Pork overall performance across four specimen-group outer folds. Values are mean ± sample SD; accuracy is expressed as percent.

Model	Accuracy (%)	Macro- Precision	Macro- Recall	Macro-F1	Macro-AUC
Spot-CNN	95.12 ± 2.14	0.9215 ± 0.0361	0.9417 ± 0.0214	0.9274 ± 0.0306	0.9970 ± 0.0020
ShuffleNetV2-0.5x	90.60 ± 2.38	0.8494 ± 0.0358	0.8562 ± 0.0460	0.8493 ± 0.0396	0.9548 ± 0.0317
EfficientNet-Lite0	92.62 ± 2.41	0.8883 ± 0.0369	0.8910 ± 0.0403	0.8845 ± 0.0392	0.9708 ± 0.0196
MobileNetV3-Small	88.93 ± 2.59	0.8373 ± 0.0287	0.8243 ± 0.0700	0.8185 ± 0.0582	0.9550 ± 0.0156
Tiny-MLP	95.00 ± 2.08	0.9235 ± 0.0332	0.9389 ± 0.0132	0.9253 ± 0.0240	0.9900 ± 0.0101

Supplementary Table S10a. Beef Sub-fresh precision, recall and F1-score across four specimen-group outer folds. Each outer-test fold contained 30 Sub-fresh image units.

Model	Precision	Recall	F1-score	Images per fold
Spot-CNN	0.8565 ± 0.0453	0.8083 ± 0.1596	0.8233 ± 0.0736	30
ShuffleNetV2-0.5x	0.6921 ± 0.0379	0.8500 ± 0.0577	0.7611 ± 0.0169	30
EfficientNet-Lite0	0.6983 ± 0.1068	0.8250 ± 0.0918	0.7550 ± 0.0972	30
MobileNetV3-Small	0.6290 ± 0.0455	0.8500 ± 0.0793	0.7229 ± 0.0581	30
Tiny-MLP	0.8455 ± 0.0646	0.9417 ± 0.0957	0.8891 ± 0.0667	30

Supplementary Table S10b. Pork Sub-fresh precision, recall and F1-score across four specimen-group outer folds. Each outer-test fold contained 30 Sub-fresh image units.

Model	Precision	Recall	F1-score	Images per fold
Spot-CNN	0.7966 ± 0.1067	0.9333 ± 0.0471	0.8548 ± 0.0495	30
ShuffleNetV2-0.5x	0.6607 ± 0.0658	0.7417 ± 0.1500	0.6936 ± 0.0908	30
EfficientNet-Lite0	0.7447 ± 0.1015	0.8167 ± 0.1453	0.7697 ± 0.0854	30
MobileNetV3-Small	0.6603 ± 0.0990	0.6500 ± 0.2472	0.6228 ± 0.1414	30
Tiny-MLP	0.8079 ± 0.1336	0.9250 ± 0.0957	0.8513 ± 0.0483	30

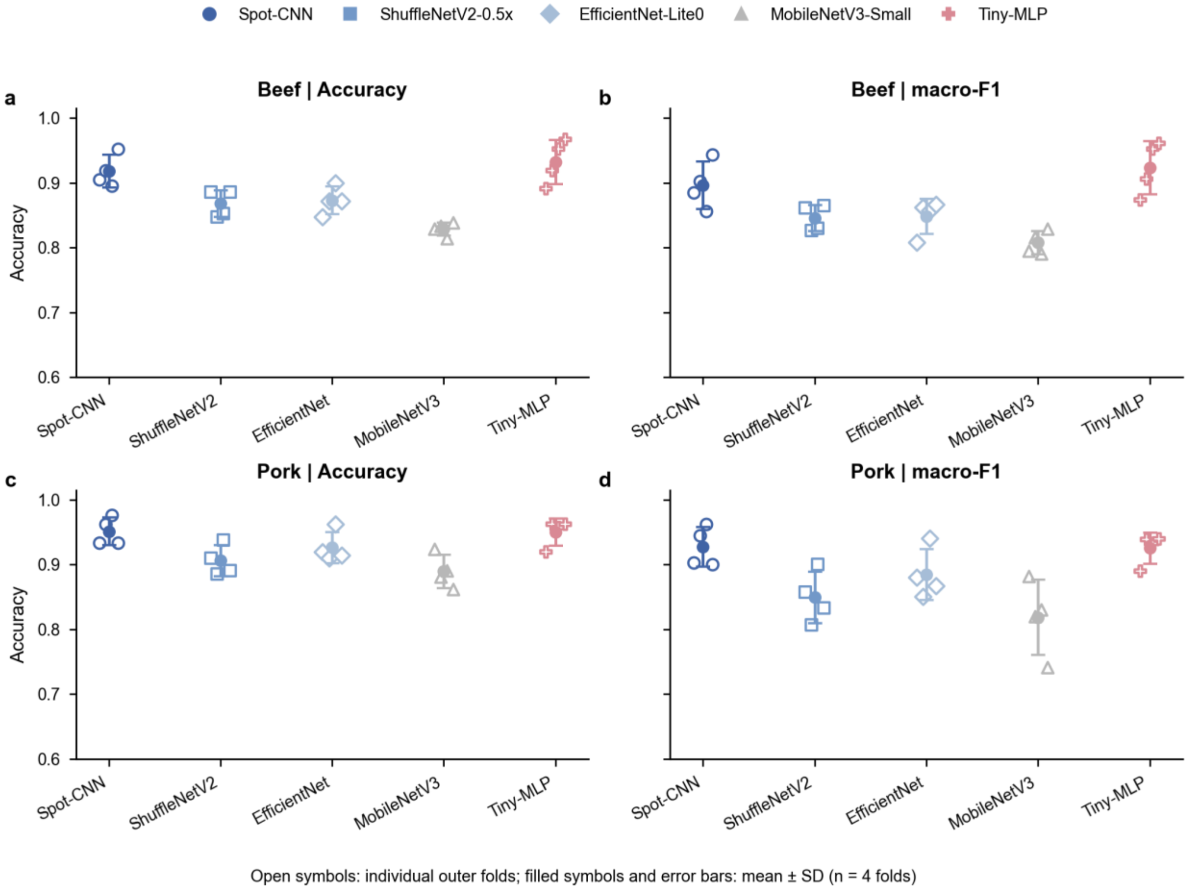

Supplementary Figure S6. Accuracy and macro-F1 of five lightweight models in four-fold specimen-group cross-validation. Beef and pork each contained 40 independently stored meat portions and used the same specimen-level grouping protocol. Open symbols indicate individual outer folds; filled symbols and error bars show mean ± sample SD.

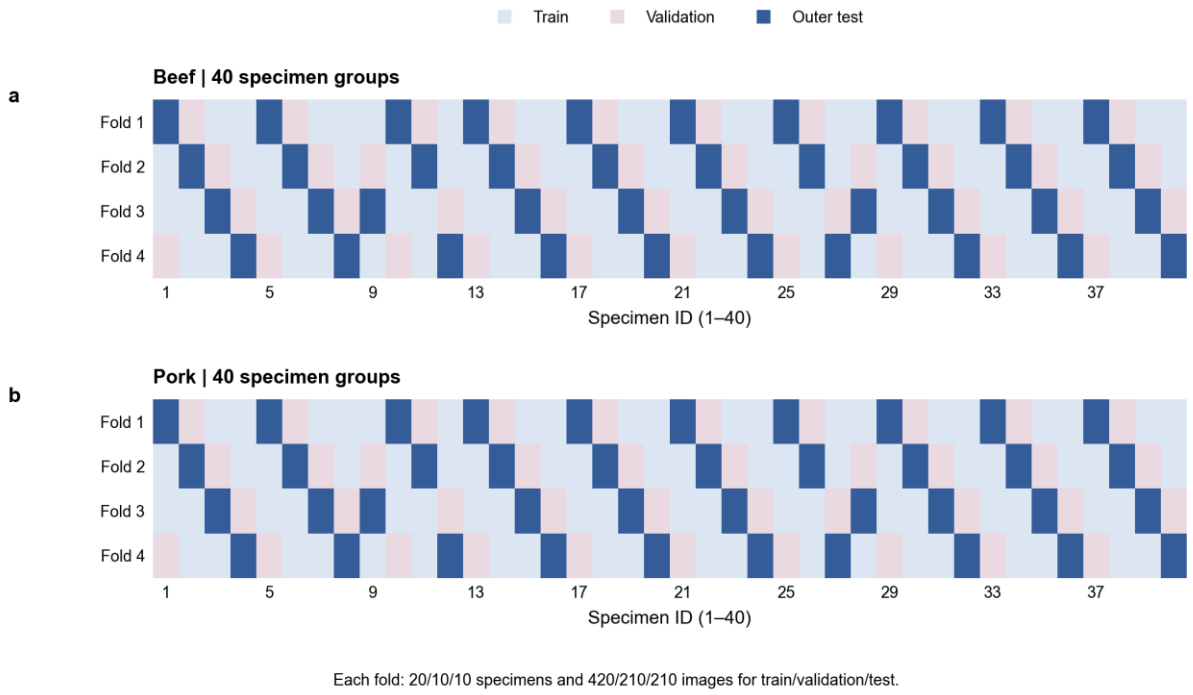

Supplementary Figure S7. Four-fold allocation of 40 beef portions (a) and 40 pork portions (b). Each row shows training, validation and outer-test roles under the same specimen-level grouping rule. Each fold contained 20, 10 and 10 portions and 420, 210 and 210 image units in the three roles, respectively.

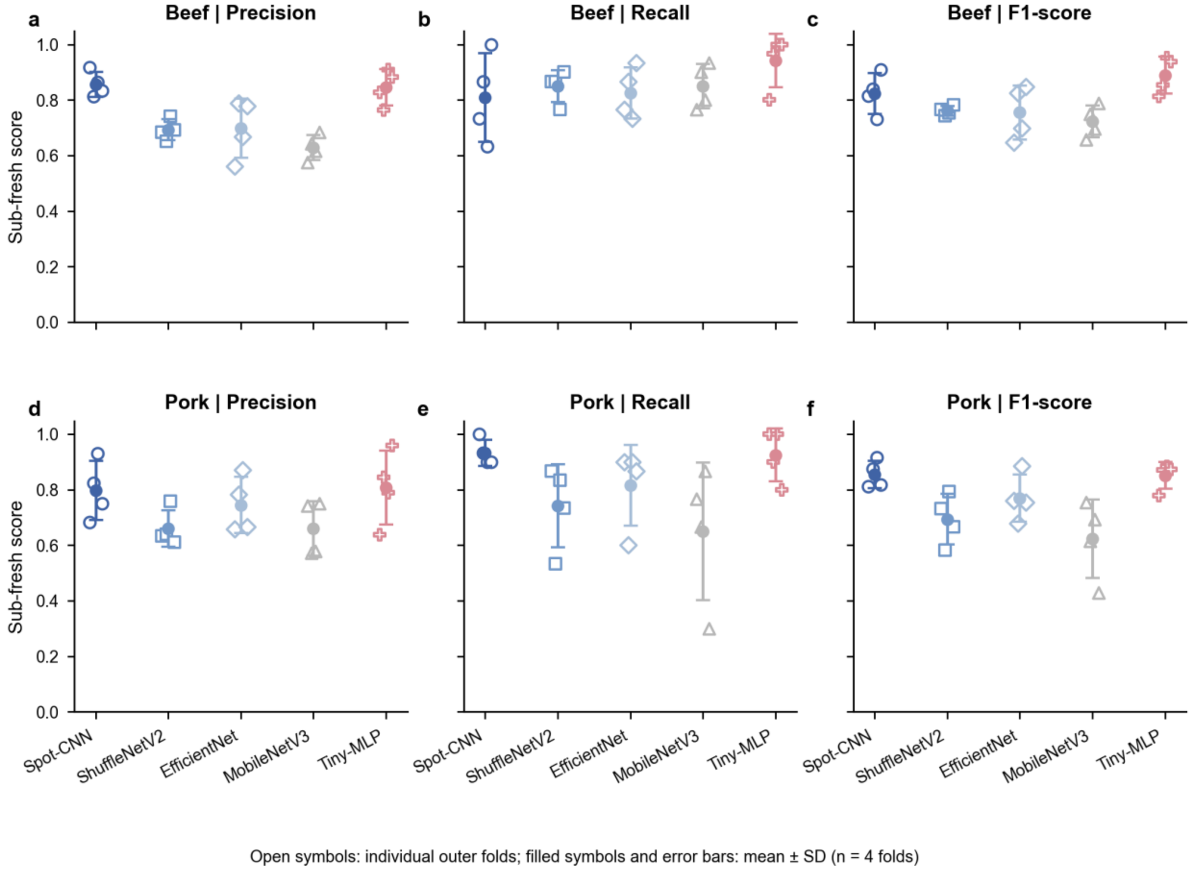

Supplementary Figure S8. Precision, recall and F1-score for the Sub-fresh class in beef (a–c) and pork (d–f). Open symbols show individual folds; filled symbols and error bars indicate mean ± sample SD across four specimen-group folds. Each outer-test fold contained 30 Sub-fresh image units from repeated observations of held-out portions.

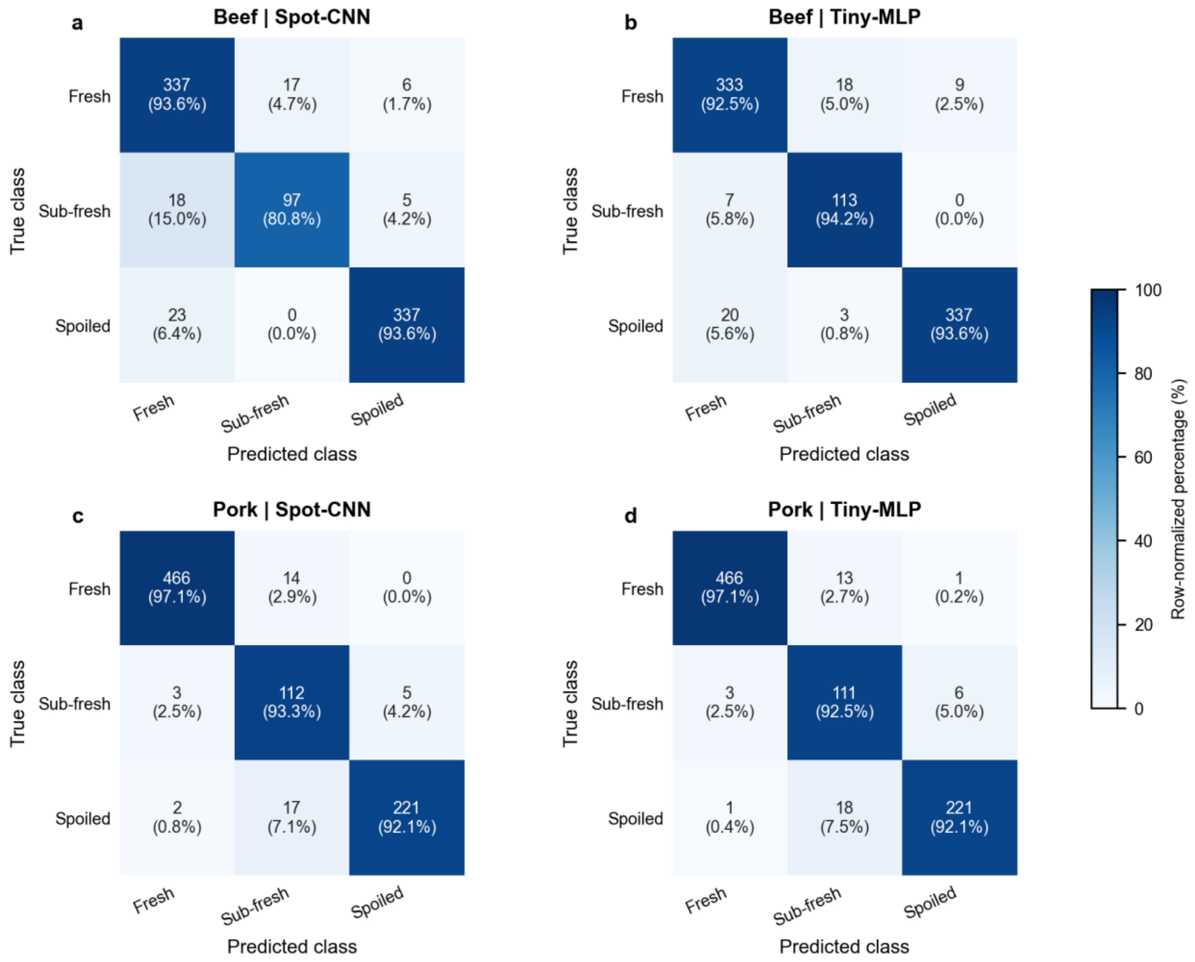

Each cell shows pooled out-of-fold count and row percentage; each image is tested once.

Supplementary Figure S9. Pooled out-of-fold confusion matrices for Spot-CNN and Tiny-MLP on beef and pork. Each image was predicted once by a model trained without its meat-portion group. Cell labels show counts and row-normalized percentages. These pooled error distributions are distinct from the mean of the four fold-level metrics.

Supplementary Table S11. Nitrogen-physisorption parameters for the measured aerogel sample. Values are from a single instrument report and are not replicate means.

Parameter	Reported value	Method or interpretation
BET specific surface area	30.6651 m^2/g	Multipoint adsorption fit
BET fitting interval	P/P_0 = 0.0503–0.2738	10 adsorption points
BET monolayer capacity	7.0478 cm^3 STP/g	Reported Vm
BET constant C	74.0	Positive fitted constant
BET correlation coefficient	r = 0.99995	Not R^2; $R^2 \approx 0.99990$
BJH adsorption cumulative pore volume	0.2691 cm^3/g	Adsorption branch
BJH desorption cumulative pore volume	0.2751 cm^3/g	Desorption branch
BJH adsorption mean pore diameter	27.8254 nm	4V/A using adsorption-branch area
BJH desorption mean pore diameter	26.3648 nm	4V/A using desorption-branch area

Supplementary portion-day aggregation tables

Supplementary Table S12. Technical-replicate prediction consistency for the fixed test split and pooled outer-test predictions from four-fold specimen-group cross-validation.

Scope	Meat	Model	Groups	Fully consistent	Consistency (%)	2:1 disagreement	3-way disagreement
Fixed test	Beef	Spot-CNN	70	62	88.6	7	1
Fixed test	Beef	ShuffleNetV2-0.5x	70	57	81.4	13	0
Fixed test	Beef	EfficientNet-Lite0	70	58	82.9	11	1
Fixed test	Beef	MobileNetV3-Small	70	52	74.3	17	1
Fixed test	Beef	Tiny-MLP	70	67	95.7	3	0
Fixed test	Pork	Spot-CNN	70	69	98.6	1	0
Fixed test	Pork	ShuffleNetV2-0.5x	70	64	91.4	6	0
Fixed test	Pork	EfficientNet-Lite0	70	69	98.6	1	0
Fixed test	Pork	MobileNetV3-Small	70	66	94.3	3	1
Fixed test	Pork	Tiny-MLP	70	70	100.0	0	0
Four-fold pooled outer test	Beef	Spot-CNN	280	265	94.6	15	0
Four-fold pooled outer test	Beef	ShuffleNetV2-0.5x	280	224	80.0	56	0
Four-fold pooled outer test	Beef	EfficientNet-Lite0	280	230	82.1	47	3
Four-fold pooled outer test	Beef	MobileNetV3-Small	280	229	81.8	49	2
Four-fold pooled outer test	Beef	Tiny-MLP	280	272	97.1	8	0
Four-fold pooled outer test	Pork	Spot-CNN	280	276	98.6	4	0
Four-fold pooled outer test	Pork	ShuffleNetV2-0.5x	280	241	86.1	37	2
Four-fold pooled outer test	Pork	EfficientNet-Lite0	280	253	90.4	27	0
Four-fold pooled outer test	Pork	MobileNetV3-Small	280	246	87.9	33	1
Four-fold pooled outer test	Pork	Tiny-MLP	280	274	97.9	6	0

For the four-fold pooled outer-test analysis, each meat type contributed 280 portion-day groups in total (70 groups per fold × 4 folds).

Supplementary Table S13. Portion-day-level performance of the five models under the fixed test split. Each test set was aggregated from 210 image units to 70 portion-day units by averaging the predicted class probabilities of the three technical replicate images.

Meat	Model	Portion-day units	Accuracy	Macro-Precision	Macro-Recall	Macro-F1	Macro-AUC
Beef	Spot-CNN	70	0.9143	0.8992	0.9333	0.9134	0.9956
Beef	ShuffleNetV2-0.5x	70	0.9000	0.8687	0.8778	0.8704	0.9922
Beef	EfficientNet-Lite0	70	0.9000	0.8659	0.9222	0.8813	0.9867
Beef	MobileNetV3-Small	70	0.8714	0.8288	0.8556	0.8365	0.9753
Beef	Tiny-MLP	70	0.9143	0.9131	0.9111	0.9114	0.9858
Pork	Spot-CNN	70	0.9429	0.9129	0.8833	0.8942	0.9809
Pork	ShuffleNetV2-0.5x	70	0.9429	0.9484	0.8667	0.8893	0.9458
Pork	EfficientNet-Lite0	70	0.9286	0.9333	0.8333	0.8519	0.9648
Pork	MobileNetV3-Small	70	0.9143	0.9231	0.8000	0.8137	0.9136
Pork	Tiny-MLP	70	0.9286	0.9333	0.8333	0.8519	0.9766

Supplementary Table S14. Portion-day-level performance across four specimen-group outer folds. Values are mean ± sample SD; accuracy is expressed as percent. Each outer-test fold contained 70 portion-day units.

Meat	Model	Portion-day units/fold	Accuracy (%)	Macro-Precision	Macro-Recall	Macro-F1	Macro-AUC
Beef	Spot-CNN	70	91.43 ± 3.09	0.9099 ± 0.0267	0.8889 ± 0.0635	0.8935 ± 0.0436	0.9888 ± 0.0070
Beef	ShuffleNetV2-0.5x	70	88.93 ± 4.10	0.8554 ± 0.0445	0.8917 ± 0.0483	0.8682 ± 0.0471	0.9786 ± 0.0119
Beef	EfficientNet-Lite0	70	89.29 ± 2.97	0.8592 ± 0.0291	0.8944 ± 0.0345	0.8710 ± 0.0308	0.9757 ± 0.0150
Beef	MobileNetV3-Small	70	84.29 ± 1.17	0.8125 ± 0.0116	0.8556 ± 0.0091	0.8240 ± 0.0094	0.9609 ± 0.0032
Beef	Tiny-MLP	70	93.57 ± 3.78	0.9185 ± 0.0430	0.9389 ± 0.0501	0.9267 ± 0.0455	0.9889 ± 0.0067
Pork	Spot-CNN	70	94.64 ± 2.14	0.9152 ± 0.0331	0.9354 ± 0.0197	0.9209 ± 0.0287	0.9973 ± 0.0021
Pork	ShuffleNetV2-0.5x	70	92.86 ± 5.08	0.8912 ± 0.0768	0.8938 ± 0.0854	0.8854 ± 0.0815	0.9639 ± 0.0316
Pork	EfficientNet-Lite0	70	93.93 ± 3.17	0.9068 ± 0.0444	0.9104 ± 0.0529	0.9022 ± 0.0495	0.9781 ± 0.0179
Pork	MobileNetV3-Small	70	90.36 ± 3.17	0.8583 ± 0.0322	0.8479 ± 0.0702	0.8447 ± 0.0549	0.9631 ± 0.0122
Pork	Tiny-MLP	70	95.00 ± 2.47	0.9242 ± 0.0413	0.9375 ± 0.0108	0.9237 ± 0.0287	0.9910 ± 0.0089

Compared with the corresponding image-unit-level results, portion-day aggregation produced limited overall changes. The maximum absolute changes under the fixed split were 2.86 percentage points in accuracy, 0.0301 in macro-F1 and 0.0290 in macro-AUC; for the four-fold mean results, the corresponding maxima were 2.26 percentage points, 0.0362 and 0.0197. The relative ranking of the leading models was not identical across all metrics: Tiny-MLP yielded the highest mean portion-day accuracy and macro-F1 for both meat types, whereas Spot-CNN retained the highest pork macro-AUC.

The branch-specific pore volumes and 4V/A mean diameters are model-derived descriptors and do not represent bulk void fraction or a uniquely defined pore-size mode. The sub-2 nm BJH outputs are displayed transparently in Figure S11 but are not used to infer a micropore-size mode. The supplied NLDFT output was not used for interpretation because its listed zeolite/cylindrical-pore model has not been validated for this composite.

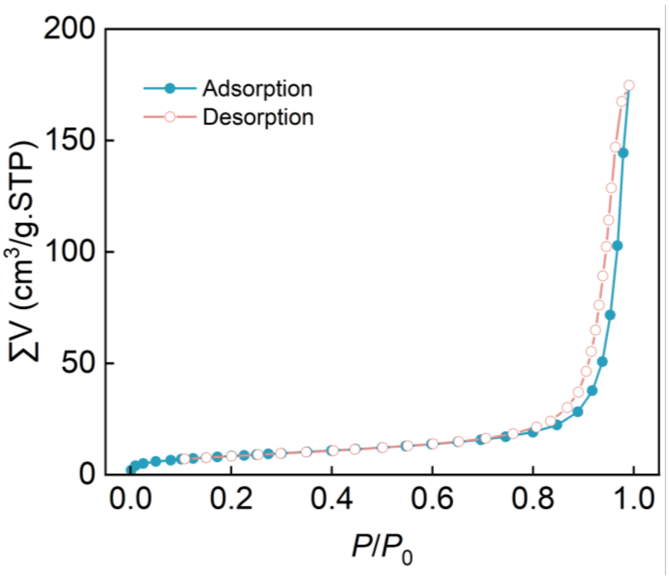

Supplementary Figure S10. N_2 adsorption–desorption isotherms.

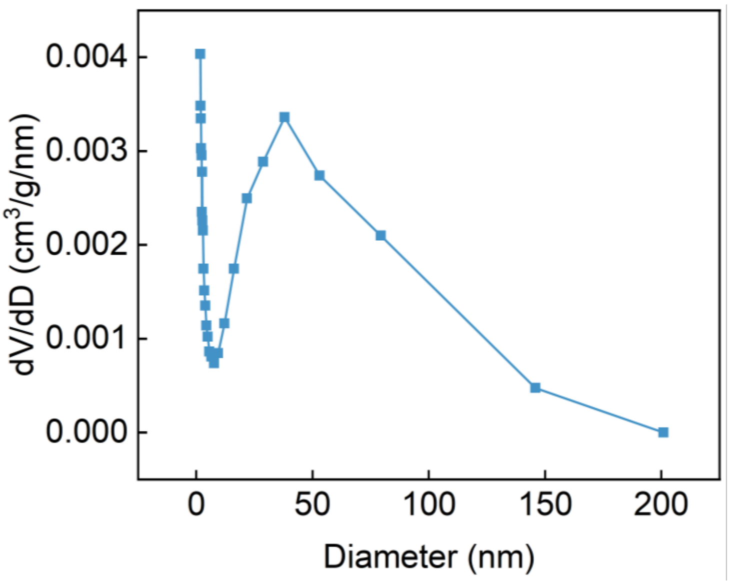

Supplementary Figure S11. Pore size distribution.

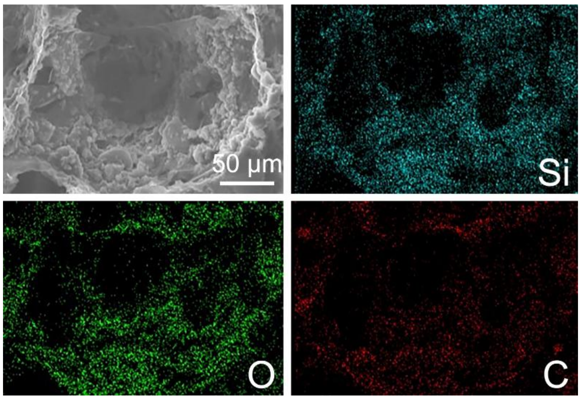

Supplementary Figure S12. SEM–EDS elemental mapping of the KGM/gelatin/starch/SiO_2 composite aerogel. The maps show Si, O and C signals within the examined region; nanoparticle agglomeration was not quantitatively assessed.
